# Small-molecule inhibitors of the CD40–CD40L costimulatory interaction are effective in pancreatic islet transplantation and prevention of type 1 diabetes models

**DOI:** 10.3389/fimmu.2024.1484425

**Published:** 2024-11-13

**Authors:** Sung-Ting Chuang, Oscar Alcazar, Brandon Watts, Midhat H. Abdulreda, Peter Buchwald

**Affiliations:** ^1^ Diabetes Research Institute, Miller School of Medicine, University of Miami, Miami, FL, United States; ^2^ Department of Surgery, Miller School of Medicine, University of Miami, Miami, FL, United States; ^3^ Department of Microbiology and Immunology, Miller School of Medicine, University of Miami, Miami, FL, United States; ^4^ Department of Ophthalmology, Miller School of Medicine, University of Miami, Miami, FL, United States; ^5^ Department of Molecular and Cellular Pharmacology, Miller School of Medicine, University of Miami, Miami, FL, United States

**Keywords:** CD154, costimulatory inhibition, immune checkpoint, immunosuppression, islet transplantation, protein-protein interaction, small-molecule drug, type 1 diabetes (T1D)

## Abstract

As part of our work to develop small-molecule inhibitors (SMIs) of the CD40-CD40L(CD154) costimulatory protein-protein interaction, here, we describe the ability of two of our most promising SMIs, DRI-C21041 and DRI-C21095, to prolong the survival and function of islet allografts in two murine models of islet transplantation (under the kidney capsule and in the anterior chamber of the eye) and to prevent autoimmune type 1 diabetes (T1D) onset in NOD mice. In both transplant models, a significant portion of islet allografts (50%-80%) remained intact and functional long after terminating treatment, suggesting the possibility of inducing operational immune tolerance via inhibition of the CD40-CD40L axis. SMI-treated mice maintained the structural integrity and function of their islet allografts with concomitant reduction in immune cell infiltration as evidenced by direct longitudinal imaging *in situ*. Furthermore, in female NODs, three-month SMI treatment reduced the incidence of diabetes from 80% to 60% (DRI-C21041) and 25% (DRI-C21095). These results (*i*) demonstrate the susceptibility of this TNF superfamily protein-protein interaction to small-molecule inhibition, (*ii*) confirm the *in vivo* therapeutic potential of these SMIs of a critical immune checkpoint, and (*iii*) reaffirm the therapeutic promise of CD40-CD40L blockade in islet transplantation and T1D prevention. Thus, CD40L-targeting SMIs could ultimately lead to alternative immunomodulatory therapeutics for transplant recipients and prevention of autoimmune diseases that are safer, less immunogenic, more controllable (shorter half-lives), and more patient-friendly (i.e., suitable for oral administration, which makes them easier to administer) than corresponding antibody-based interventions.

## 1 Introduction

Immune checkpoints (ICPs), critical regulators of immune responses, involve cell surface protein-protein interactions (PPIs) that can either inhibit or stimulate T cell mediated immune reactions; therefore, ICP modulation has considerable therapeutic potential for treatment of cancer on one hand and for treatment of autoimmune diseases on the other (1–3). Coinhibitory or costimulatory PPIs belong to two main families: the immunoglobulin superfamily (IgSF), e.g., CD28–CD80/86, CTLA4(CD152)–CD80/86, ICOS(CD278)–ICOS-L(CD275), and PD-L1(CD274)–PD-1(CD279) and the TNFR–TNF superfamily (TNFSF), e.g., CD40–CD40L(CD154), OX40(CD134)–OX40L(CD252), and 4-1BB(CD137)–4-1BB-L. The development of ICP inhibitors that target coinhibitory interactions, such as CD80–CTLA4 and PD-1–PD-L1, to overcome immunological anergy and invigorate the T effector cells restoring their ability to destroy cancer cells was named “Breakthrough of the Year” in 2013 by *Science* magazine (4). The subsequent development of immuno-oncology with the introduction of antibodies against PD-1 (nivolumab, pembrolizumab), PD-L1 (atezolizumab, durvalumab, and avelumab), and CTLA4 (ipilimumab) has led to a paradigm shift in the treatment of cancer (5).

The therapeutic applicability of biologics such as antibodies is, however, often hindered by their immunogenicity, which is elicited by their protein nature (6), and exacerbated by their long elimination half-lives (typically two to three weeks (7)), which makes it difficult to rapidly abolish unwanted side effects when they occur (8). Not surprisingly, antibodies tend to have more post-market safety issues than traditional small-molecule drugs (9). Moreover, the use of immunomodulatory biologics is further complicated by the high likelihood of unwanted adverse reactions that include cytokine release syndrome, serious and protracted infections, malignancy, and anaphylaxis among others (10). Biologics-based cancer immunotherapies have been found to cause immune-related adverse events (irAEs) in a large fraction of treated patients, sometimes as high as 50% (11). In general, small molecules may represent viable, safer alternatives that can also be orally bioavailable. This is particularly important for prospective preventive therapies, for example in type 1 diabetes (T1D), because such therapies have to be patient-friendly (12) to encourage the adherence and compliance needed for such long-term treatments to achieve efficacy (13, 14).

However, the modulation of PPIs with small molecules can be challenging because the corresponding protein interfaces lack well-defined binding sites such as those typically needed for the strong binding of such small-molecule ligands. Nevertheless, during the last two decades, tens of PPI-targeting small-molecule inhibitors (SMIs) reached preclinical development (15–22), and three are already approved by the FDA for clinical use: • venetoclax (ABT-199; Venclexta^®^/Venclyxto^®^), an inhibitor targeting PPIs in the B cell lymphoma 2 (BCL-2) family approved in 2015 for the treatment of chronic lymphocytic leukemia (CLL), small lymphocytic lymphoma (SLL), and acute myeloid leukemia (AML) (23); • lifitegrast (SAR 1118; Xiidra^®^), an LFA-1–ICAM-1 inhibitor approved in 2016 for the treatment of dry eye (24); and • fostemsavir (BMS-663068; Rukobia^®^), a prodrug of temsavir that blocks gp120–CD4 binding approved in 2020 as an antiretroviral for adults living with HIV/AIDS (25). Such SMIs of PPIs (SMIPPIs) can lead to novel therapies that, compared to antibodies, are less immunogenic, more controllable (due to their shorter half-lives), easier to administer and thus more patient-friendly (by virtue of being suitable for oral administration), and more cell- and tissue-permeable (due to their better biodistribution properties). Notably, even in the field of TNF-targeting therapeutics, where the treatment of rheumatoid arthritis (RA) has been revolutionized by the use of anti-TNF biologics, such as etanercept, infliximab, adalimumab, certolizumab pegol, and golimumab, which have been considered among the most transformative drugs of the 1985–2010 period (26), there is increasing interest in SMIs. One such SMI developed by Sanofi, SAR441566, is currently in Phase 2 clinical trials for RA treatment (e.g., NCT06073093) (27, 28).

Along these lines, we focused on identifying SMIs of the CD40(TNFRSF5) – CD40L(CD154, TNFSF5) costimulatory PPI (29) because blockade of this ICP has been consistently shown to be a highly effective immunomodulatory therapy in general (29–34) and for pancreatic islet transplantation in particular. For the latter, CD40–CD40L inhibition allowed the engraftment of allogeneic (and sometimes even xenogeneic) islets resulting in long-term insulin independence with the possibility for induced operational tolerance in various mouse (35–45) and nonhuman primate (NHP) allo-transplant models (46–52). Furthermore, CD40L inhibition has shown great therapeutic promise in the prevention of autoimmune diseases (29, 31, 34, 53) including T1D (54–61). It has been suggested that targeting the CD40–CD40L PPI may provide “the next major novel class of costimulatory inhibitors to treat autoimmune disease” (62). Notably, with the realization that the thromboembolic complications encountered with the first-generation of CD40L antibodies like ruplizumab (hu5c8) were driven by their Fc region and can be avoided (20, 63–70), there is a resurgence of interest in CD40–CD40L blockade as exemplified by the title of a recent review: “*Phoenix from the flames: Rediscovering the role of the CD40-CD40L pathway*…” (71). Indeed, second-generation Fc-silent anti-CD40L antibodies retain immunomodulatory activity but do not activate platelets (71–74). Examples include • letolizumab (BMS-986004; Bristol-Myers Squibb) for the treatment of immune thrombocytopenic purpura (ITP) and graft versus host disease (GVHD) (73); • dapirolizumab pegol (CDP7657; UCB Pharma) for systemic lupus erythematosus (SLE) – currently in Phase 3 clinical trials (75, 76); • frexalimab (SAR441344, INX-021; Sanofi) for multiple sclerosis (MS) – currently in Phase 2 trials (77); • tegoprubart (AT-1501; Eledon) for amyotrophic lateral sclerosis (ALS) – currently in Phase 2a trials (78); • TNX-1500 (Tonix), which has shown efficacy in NHP transplant models (79); and • dazodalibep (HZN-4920, VIB4920; Viela Bio, MedImmune, and Horizon Therapeutics) to prevent autoimmune RA – currently in Phase 2 studies (80, 81), as well as Sjögren’s disease – currently in Phase 2 with very promising results (82). Tegoprubart has also shown efficacy in kidney and islet transplants in NHPs (83) and early clinical trials. Iscalimab (CFZ533), a human Fc-silenced, non-depleting, IgG1 anti-CD40 monoclonal antibody was shown to be effective in Sjögren’s disease in a large Phase 2b study conducted at 71 sites in 23 countries (84). Thus, the CD40–CD40L interaction remains an attractive therapeutic target in islet transplant applications and for the prevention of T1D.

We have designed and developed SMIs for the CD40–CD40L PPI (85, 86), explored structure-activity relationships for more than 50 newly synthesized compounds, and identified lead SMIs that showed CD40L-inhibitory activity as well as selectivity *in vitro* and *in vivo* (87, 88). We now report further long-term *in vivo* studies showing that two of our most promising CD40–CD40L SMIPPIs, DRI-C21041 and DRI-C21095, were able to • significantly prolong the survival of islet allografts in two mouse models of islet transplantation in different sites and • prevent the onset of autoimmune diabetes in the non-obese diabetic (NOD) mouse model of T1D.

## 2 Materials and methods

### 2.1 Drugs

DRI-C21041 and DRI-C21095 were synthesized either in-house or by WuXi AppTec (Kowloon, Hong Kong) as described before in detail (87, 88). Structures were confirmed by high-resolution mass spectrometry (HRMS) and NMR; purities were confirmed to be ≥95% by HPLC. The anti-CD40L mouse antibody (clone MR-1) was purchased from Bio X Cell (Lebanon, NH, USA).

### 2.2 Animal care and treatment

Mice used for these studies were obtained from Jackson Laboratories (Bar Harbor, ME, USA). The following strains were used: NOD (NOD/ShiLtJ; strain # 001976), C57BL/6 (C57BL/6J; strain # 000664), and DBA/2 (DBA/2J; strain # 000671). All animal studies were reviewed and approved by the University of Miami Institutional Animal Care and Use Committee (IACUC). Procedures were conducted according to the guidelines of the Committee on Care and Use of Laboratory Animals, Institute of Laboratory Animal Resources (National Research Council, Washington, DC, USA). Animals were housed in micro-isolated cages with free access to autoclaved food and water in Virus Antibody Free (VAF) rooms managed by the Department of Veterinary Resources of the University of Miami.

### 2.3 Prevention of diabetes onset in NOD mice

For the diabetes prevention studies, young female NOD mice (*n* = 5/group) were subcutaneously (s.c.) treated twice daily (b.i.d.) between the ages of 5 and 18 weeks with DRI-C21095 and DRI-C21041 at 20 and 12.5 mg/kg, respectively, administered in 20% hydroxypropyl-β-cyclodextrin (HPβCD) solution as vehicle. A corresponding control group was treated with vehicle only. Mice were monitored for diabetes onset by glycosuria once a week starting from week 10 until 40 weeks of age. Once hyperglycosuria was detected, monitoring was switched to daily glycemia measurements using portable glucometers (Contour Next; Bayer, Mishawaka, USA). Animals with three consecutive readings of blood glucose (BG) >300 mg/dL were considered diabetic and humanely euthanized for tissue analysis. Animals remaining normoglycemic by week 40 of age were euthanized shortly thereafter and blood as well as pancreas, spleen, lymph nodes, and other organs were collected for various analyses.

### 2.4 Immunophenotyping

Mouse splenocytes were collected by mashing spleens through a 70 μm cell strainer, subjected to ACK lysis to remove erythrocytes, and resuspended in Hank’s buffer. Cells were diluted in trypan blue and counted in a hemocytometer. One million viable cells were incubated with Live and Dead solution (Zombie UV, BioLegend, San Diego, CA, USA; cat. no. 423107) in PBS for 20 min at 4°C. Cells were washed and incubated with universal Fc blocker containing anti-mouse CD16/32 (clone 2.4G2) for 20 min at 4°C. Then, cells were incubated for 30 min at room temperature (RT) protected from light in a master mix for surface staining by the following fluorescence-labeled antibodies against NK-1.1 (BD Biosciences, Franklin Lakes, NJ, USA; cat. no. 741715); CD62L (BioLegend; cat. no. 104450); CD8a (BD Biosciences; cat. no. 553036); CD44 (BD Biosciences; cat. no. 562464); CD25 (BioLegend; cat. no. 102016); CD4 (BioLegend; cat. no. 100492); and CD3 (Invitrogen, Carlsbad, CA, USA; cat. no. 56-0032-82) at dilutions according to the recommendations of the suppliers. Cells were washed twice in PBS buffer supplemented with 1% BSA (VWR/Avantor, Radnor, PA, USA; cat. no. 0332), incubated with FIX/PERM (Thermo-Fisher Scientific, Waltham, MA, USA; kit cat. no. 00-5523-00) for 30 min at 4°C, and then washed twice with PERM buffer (Thermo-Fisher Scientific; kit cat. no. 00-5523-00);. Afterwards, cells were subjected to intracellular staining with anti-FoxP3 (Invitrogen; cat. on. 15-5773-82), in PERM buffer for 30 min at 4°C, then washed twice with wash buffer (1% BSA in PBS). After centrifugation, wash buffer was added to resuspend the cells before analysis. Single-color compensation samples for flow cytometry were prepared using the same antibodies with UltraComp eBeads (Thermo Fisher Scientific; cat. no. 01-3333-42). Data were acquired using a Cytek Aurora flow cytometer (Cytek, Fremont, CA, USA) and analyzed with Kaluza software (Beckman Coulter, Brea, CA, USA).

### 2.5 Islet isolation, transplantation in the kidney of C57BL/6 mice, and treatment

Pancreatic islets used for transplantation were obtained by enzymatic digestion of DBA/2 donor mouse pancreata, followed by purification on density gradients using protocols standardized at the Preclinical Cell Processing and Translational Models Core of our institute (Diabetes Research Institute, DRI) (37, 40). After overnight culture, isolated islets were implanted in fully anesthetized male and female recipient C57BL/6 mice under the kidney subcapsular space, as previously described (37, 40). Prior to transplantation, diabetes was induced with a single dose of streptozotocin (STZ) treatment (200 mg/kg, i.v.) and mice confirmed to be hyperglycemic (i.e., three consecutive non-fasting BG readings > 300 mg/dL) were transplanted with 500 islet equivalents (IEQ) under the kidney capsule. DRI-C21095 and DRI-C21041 treatments (10, 12.5, 15, and 20 mg/kg) were administered s.c. in 20% HPβCD vehicle twice daily (b.i.d.) starting the day before transplant (day -1) until day 30 post-transplant. Mice treated with anti-CD40L antibody (MR-1) received 250 μg administered once a day (s.c.) on days -1, 0, 3, and 7. Transplanted mice remaining normoglycemic at 60 days post-transplant underwent unilateral nephrectomy of the graft-bearing kidney to confirm function of the islet graft and were euthanized shortly thereafter.

### 2.6 *In vivo* imaging of islets transplanted in the ACE of C57BL/6 mice and treatment

In these studies, normoglycemic C57BL/6 mice were transplanted with allogeneic DBA/2 donor islets in the anterior chamber of the eye (ACE). Recipient mice were not pretreated with STZ since the ACE-islet grafts are monitored directly through the transparent cornea. The recipients also expressed green fluorescent protein (GFP) in either macrophages or CD8 T cells; these mice were generated by crossing FVB-Tg(Csf1r-icre)1Jwp/J (strain # 021024; Jackson Laboratories) or C57BL/6-Tg(Cd8a-cre)1Itan/J (strain # 008766; Jackson Laboratories), respectively, with B6.Ai38(RCL-GCaMP3)^lox/lox^ (strain # 014538; Jackson Laboratories) and breeding for >10 generations to reach homozygosity. Homozygous mice received 50 IEQs in one eye (DBA/2 islets), as previously described in detail (89). Five to seven mice were used per treatment group. MR-1 treatment was as described above. Mice treated with DRI-C21095 (20 mg/kg) or DRI-C21041 (12.5 mg/kg) received s.c. injections twice daily (b.i.d.) in 20% HPβCD vehicle up to day 60 post-transplantation. The structural integrity of ACE-transplanted islets was longitudinally monitored by direct visualization and their survival was assessed by quantitative analysis of individual islets volume, as previously described in detail (52, 89–92). In brief, islets engrafted on top of the iris were mapped in digital images of the eye acquired during the first week after transplantation and revisited during the repeated imaging sessions in these longitudinal studies. The islets were imaged noninvasively through the cornea by confocal microscopy using the 633 nm laser backscatter (reflection) (90), and the islet-infiltrating immune cells (macrophages and T cells) were visualized based on the GFP expression. Three-dimensional (3D) confocal micrographs were acquired using a 20× water immersion objective in *z*-stacks spanning the full height of the ACE-transplanted islets, and analysis of the individual islet volume was performed in the 3D images. Survival of individual islets was defined when the individual islet maintained its volume above 70% relative to its corresponding baseline (acquired during the first week after transplantation). Immune cell infiltration into the islets was quantified based on the GFP volume inside individual islets and expressed as the percentage of the corresponding islet volume. Volumetric analysis in the images was performed using Volocity software version 6.3.1 (Quorum Technologies Inc.; Puslinch, ON, Canada).

### 2.7 Immunofluorescence

Harvested tissues were immediately placed in a 15 mL Falcon tube containing 4% paraformaldehyde (PFA), incubated at room temperature (RT) for 4 h, and washed three times with PBS for a total of 15 min. They were transferred into a tube containing 30% (w/v) sucrose solution and incubated at 4°C overnight. Eyes were then mounted in Tissue-Tek O.C.T. (Sakura Finetek, Torrance, CA, USA) and snap frozen by placing the tissue mold on dry ice. Tissue sections (14 μm thick) were collected on glass slides and kept at –80°C until analysis. For immunofluorescence staining, samples were subjected to antigen retrieval by standard protocol, washed three times with PBS, and permeabilized for 1 h at RT with 0.3% Triton X-100 in 10% Fc Block (SuperBlock Blocking Buffer, Pierce, Rockford, IL, USA) or normal goat serum. After blocking for at least 1 h, samples were incubated overnight at 4°C with anti-insulin (guinea pig, 1:500; Dako, Carpinteria, CA, USA) and anti-glucagon (rabbit, 1:500; Abcam, Cambridge, MA, USA) primary antibodies. Sections were washed 5 times with PBS plus 0.05% Tween 20 and then incubated with the secondary antibodies: goat anti-rabbit AlexaFluor 488 and goat anti-guinea pig AlexaFluor 647 (both at 1:200; Life Technologies, Carlsbad, CA, USA). Nuclear counterstain was performed by DAPI (1:2000; Life Technologies). Slides were washed another 5 times with PBS plus 0.05% Tween 20 and 2 times with PBS alone before applying mounting media (Clear-MOUNT with TRIS, Electron Microscopy Science, Hatfield, PA, USA) and cover slips. Immunofluorescence imaging was performed using a Leica Stellaris 5 confocal microscope (Leica, Deerfield, IL, USA).

### 2.8 Pharmacokinetic evaluation in mice

Pharmacokinetics (PK) in mice was evaluated by quantifying plasma concentration in blood samples (50 μL) drawn from the orbital sinus at predefined time points (0, 0.5, 1, 2, 4, 6, and 24 h) following administration of a single dose (30 mg/kg, s.c.) to C57BL/6 mice (*n* = 4; 10-week-old males and females). Plasma concentrations were obtained using LC/MS performed at the Mass Spectrometry Laboratory, Department of Chemistry, University of Florida (Gainesville, FL, USA) on a ThermoFinnigan, LTQ X mass spectrometer with a ThermoScientific Hypersil GOLD aQ (150 x 2.1 mm, 3 μm) column (Thermo Fisher Scientific, Waltham, MA, USA) using mobile phases containing 0.1% formic acid in water and 0.1% formic acid in acetonitrile. Concentrations were calculated from the corresponding peak areas following a calibration curve in the 0.8 to 100 μg/mL range. Experimental concentration data obtained this way were fitted with a standard one-compartment, first-order absorption, first-order elimination PK model (87, 93) implemented as a custom equation in GraphPad Prism (GraphPad, La Jolla, CA, USA; version 10.1.2) to estimate the elimination rate constant *k*_10_ and the corresponding elimination half-life, *t*_1/2_ = ln(2)/*k*_10_.

### 2.9 Statistics and data fitting

The percent of functioning islet allografts in the transplantation models and the diabetes-free survival of NOD mice in the T1D prevention model were shown as Kaplan-Meier survival plots with asterisks denoting statistically significant differences versus the vehicle-treated group as determined by the log rank (Mantel-Cox) test using GraphPad Prism (GraphPad; version 10.1.2) (^*^ *p* < 0.05, ^**^ *p* < 0.01).

## 3 Results

### 3.1 Prolongation of survival and function of islet allografts transplanted in the kidney subcapsular space

**Figure 1:**
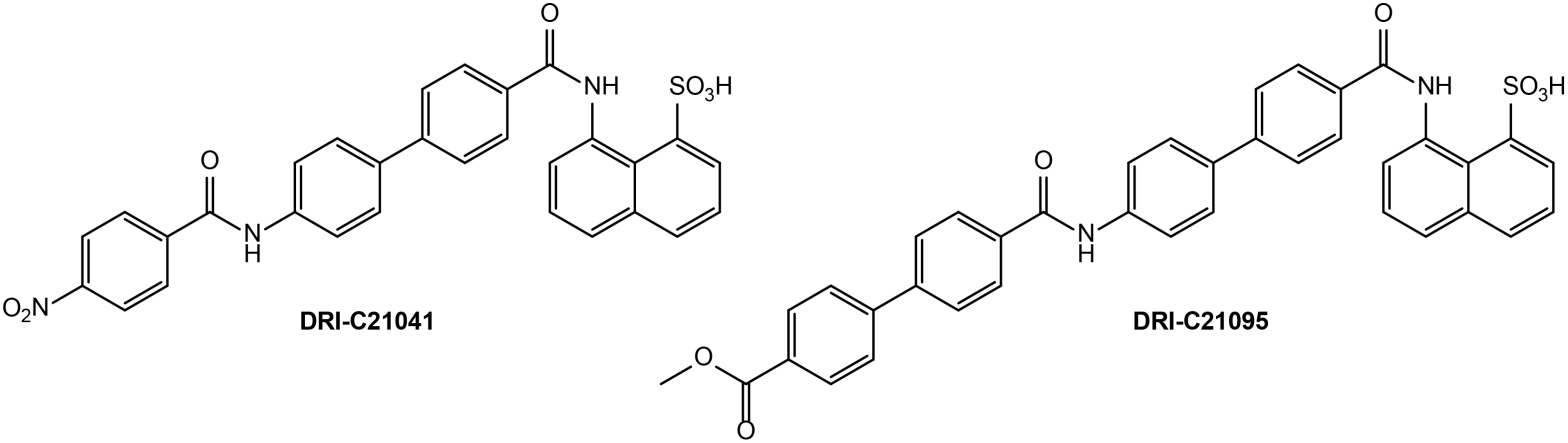
Chemical structures of DRI-C21041 and DRI-C21095, the SMIPPI compounds evaluated in the present study.

**Figure 2:**
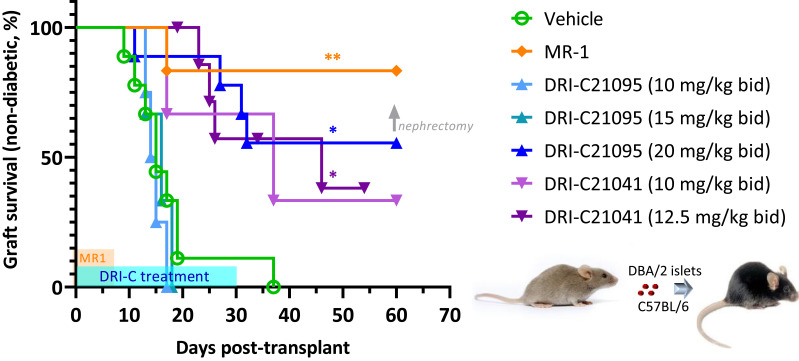
DRI-C21041 and DRI-C21095 prolong survival of islet allografts in the kidney subcapsular space of full MHC-mismatched recipients. Islets isolated from DBA/2 donors were transplanted under the kidney capsule of STZ-induced diabetic C57BL/6 mice (500 IEQ). DRI-C21095 and DRI-C21041 were administered s.c. at doses of 10–20 mg/kg (b.i.d.) in 20% HPβCD vehicle. DRI-C treatments in separate groups started on day -1 and continued until day 30, as shown in light blue on the time axis. Control mice treated with the vehicle received treatment during the same period. A positive control group was treated with anti-CD40L monoclonal antibody (mAb; clone MR-1) at a daily dose of 250 μg/mouse (~10 mg/kg) on days -1, 0, 3, and 7, as indicated by the orange-colored box along the time axis. Nephrectomy on day 60 in DRI-C treated mice maintaining normoglycemia (non-diabetic) confirmed the graft function upon return to hyperglycemia after removal of the islet graft-bearing kidney (BG > 300 mg/dL). Asterisks denote statistically significant differences versus the vehicle-treated group as determined by log rank (Mantel-Cox) test (^*^ *p* < 0.05, ^**^ *p* < 0.01; *n* = 3–9 mice).

We evaluated the immunosuppressive efficacy of our SMIs targeting the CD40–CD40L PPI (DRI-C21041 and DRI-C21095; **Figure 1**) in an allogeneic islet transplantation model with full MHC-mismatch between donors and recipients. DBA/2 donor islets (MHC Class-I H-2K^d^) were transplanted under the kidney capsule of STZ-induced diabetic C57BL/6 (MHC Class-I H-2K^b^) mouse recipients that were then treated with various drug regimens. The ability of these recipients to maintain normoglycemia following the transplant was assessed via monitoring of blood glucose levels. DRI-C21095 and DRI-C21041 were administered at doses of 10, 12.5, 15, and 20 mg/kg (b.i.d., s.c.) from days -1 to 30 after transplant. Matched control mice treated with vehicle only (20% HPβCD) or the mouse anti-CD40L monoclonal antibody (MR-1; on days -1, 0, 3, 7 after transplant) were included as negative and positive controls, respectively (**Figure 2**). These studies revealed a noticeable dose-dependency: the two lowest doses of DRI-C21095 produced no effect, whereas the highest doses of both compounds produced significant prolongation of graft survival and function in 40%–50% of recipients even well after termination of treatment in a manner similar to the positive control MR-1 antibody. Unilateral nephrectomy of the graft-bearing kidney in DRI-C treated mice that remained non-diabetic (normoglycemic) as of day 60 post-transplant resulted in these mice becoming hyperglycemic (BG > 300 mg/dL), thereby confirming the sustained survival and function of the islet allografts in association with the DRI-C treatments.

### 3.2 Prolongation of survival of islet allografts transplanted in the ACE

**Figure 3:**
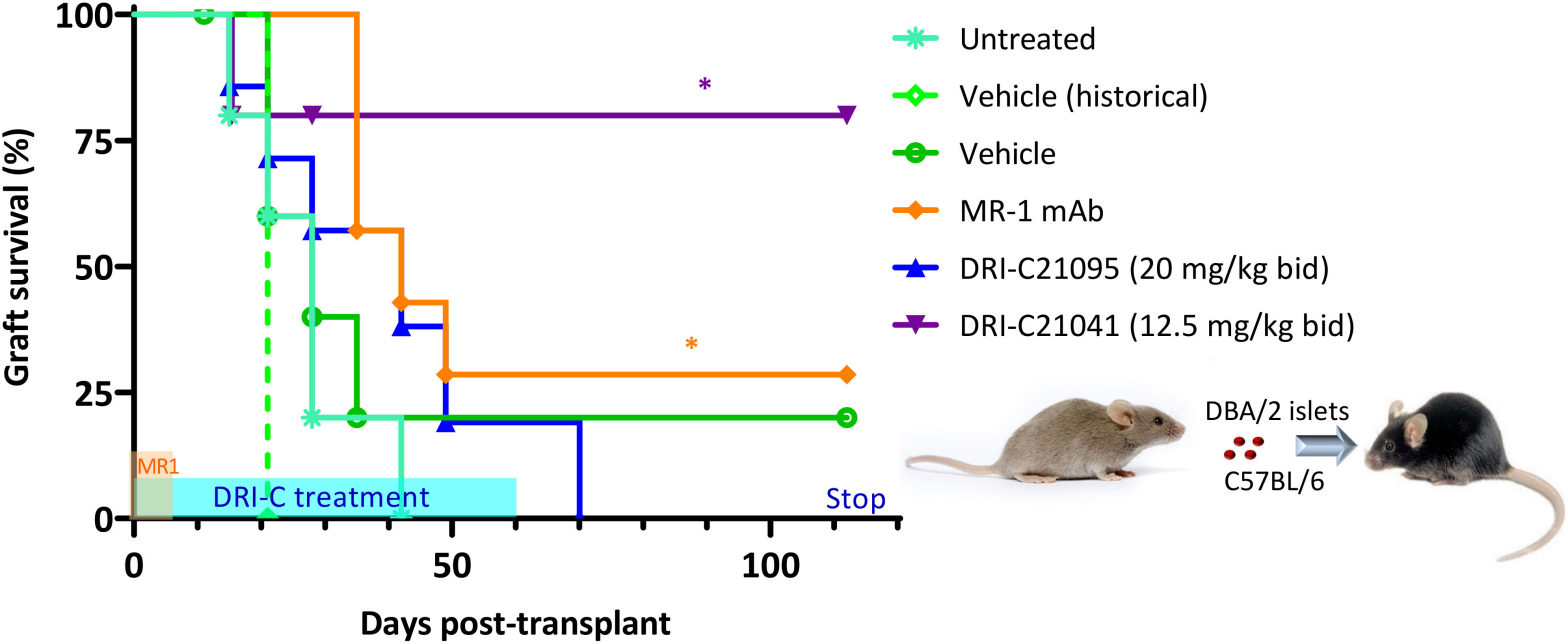
DRI-C21041 and DRI-C21095 prolong survival of islet allografts in the ACE model. Islets isolated from DBA/2 donors were transplanted into the anterior chamber of the eye (ACE) of C57BL/6 mice treated with DRI-C21095 (20 mg/kg, b.i.d.), DRI-C21041 (12.5 mg/kg, b.i.d.) – both s.c. in 20% HPβCD vehicle up to day 60, vehicle only, and MR-1 anti-CD40L mAb (250 μg/mouse, ~10 mg/kg, on days -1, 0, 3, 7 of transplant). Survival of the ACE-transplanted islet allografts was assessed by longitudinal volumetric analysis, as detailed in Methods. Most mice treated with DRI-C21041 did not reject their allografts long-term, even after treatment was stopped, until the end of follow-up (day 115); see **Figures 4A–C** for longitudinal islet images and volume analysis. Asterisks denote statistically significant differences versus the untreated group, log rank (Mantel-Cox) test (^*^ *p* < 0.05; *n* = 5–7 mice).

**Figure 4:**
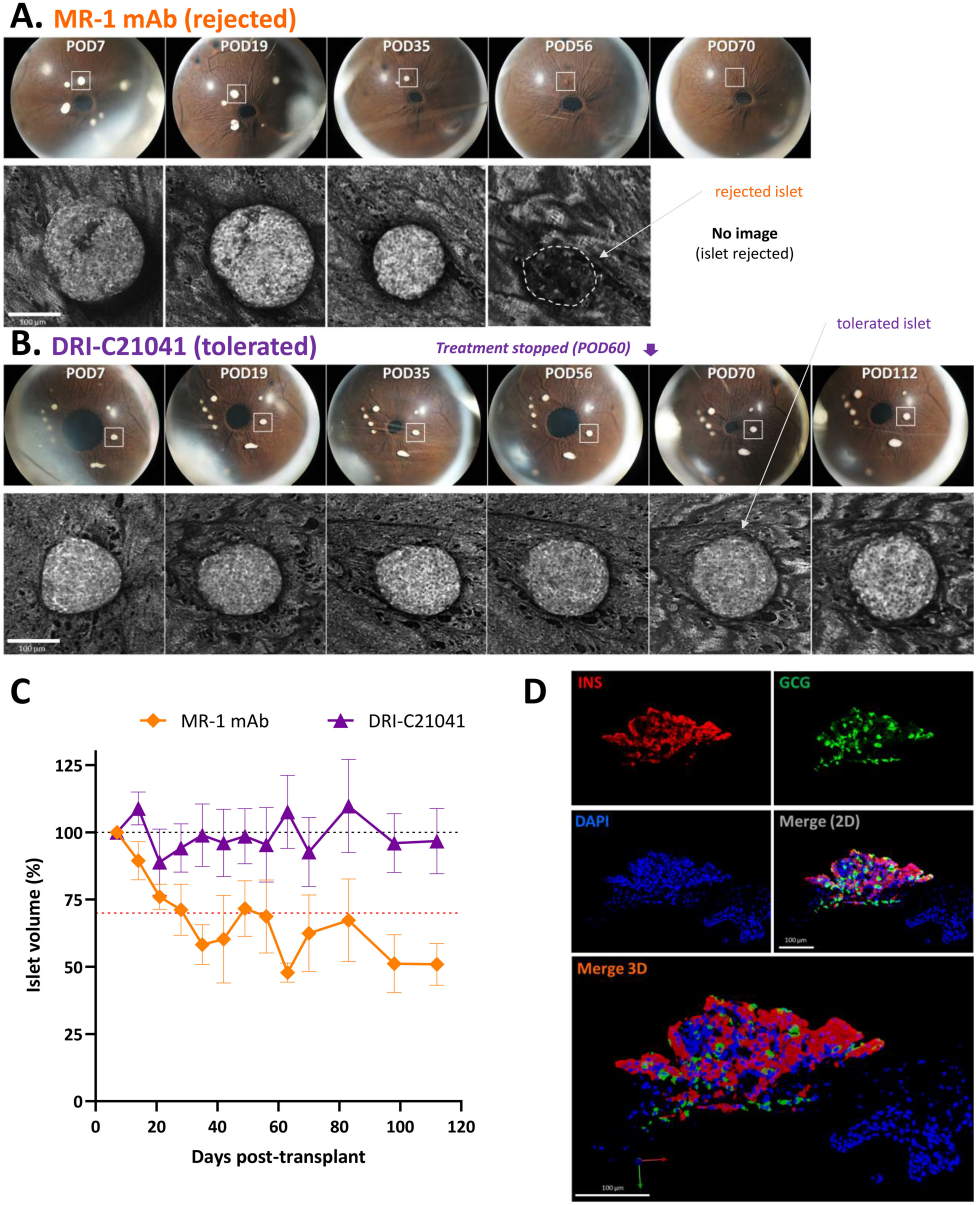
Imaging of ACE-transplanted islet grafts and quantification of their structural integrity over time. **(A, B)** Direct noninvasive and longitudinal imaging of ACE islets. Representative longitudinal images of ACE-transplanted DBA/2 islets in C57BL/6 mouse recipients treated either with MR-1 mAb **(A)** or DRI-C21041 (12.5 mg/kg b.i.d.) **(B)** from the study shown in **Figure 3**. Images showing the whole eye (top rows) with the islet highlighted in the longitudinal confocal micrographs (lower rows) marked with a white square. The shown MR-1–treated mouse rejected its islets by day 56 post-transplant (POD = post-operative day) and the DRI-C21041–treated mouse tolerated its islets until the end of the follow-up. The change in the structural integrity and volume of the rejected islet in the MR-1 treated mouse was evident, whereas islets in the DRI-C21041 treated mouse maintained their integrity and volume until the end of follow-up (last day of imaging, POD112). See C for overall longitudinal changes in the islet volume in MR-1– and DRI-C21041–treated mice. **(C)** Structural integrity of islet allografts is maintained in DRI-C21041 treated mice. Average islet volume per treatment group for DRI-C21041 and MR-1 mAb shown as determined by *in vivo* volumetric analysis in 3D confocal *z*-stacks of the individual islets and normalized to baseline (means ± SEM for *n* = 19–22 islets from 3–4 mice per group). **(D)** Fluorescence confocal micrographs confirming positive insulin and glucagon immunostaining in tolerated ACE-transplanted islet allografts in DRI-C21041 treated recipients. Representative immunofluorescence images of an ACE-transplanted DBA/2 islet in a section of the eye of a C57BL/6 recipient mouse that was treated with DRI-C21041 (12.5 mg/kg, b.i.d., s.c. up to day 60) showing positive immunostaining for insulin (red) and glucagon (green), thus, further confirming function of the islet allograft until the end of the follow-up on day 115 after the transplant. DAPI nuclear counterstain was used (blue), and slides were imaged at 40× magnification.

We also evaluated the immunosuppressive efficacy of our SMIPPIs in a different model of islet transplantation using the anterior chamber of the eye (ACE) as transplant site (52, 89–92). DRI-C21095 (20 mg/kg) and DRI-C21041 (12.5 mg/kg) were administered (b.i.d., s.c.) starting on the day of transplant (day 0) and maintained until day 60 post-transplant (**Figure 3**). As before, DBA/2 mice were used as islet donors and C57BL/6 mice as recipients to maintain full MHC-mismatch. In these studies, the recipients were normoglycemic and expressed GFP in either macrophages or T cells – this allowed direct *in vivo* visualization of these cells and the tracking of their infiltration into the ACE-transplanted islets. Survival of the islet allografts was monitored via longitudinal noninvasive imaging and quantitative volume analysis, taking advantage of the transparency of the transplant site (**Figures 4A–C**). In this model, DRI-C21041 was particularly effective: 80% of recipients maintained the structural integrity and function of their islet allografts up to the end of follow-up (115 days post-transplant), significantly longer than did the vehicle-treated and untreated control mice that rejected their grafts with a median of 28 days (**Figure 3**). Rejection in the controls was similar to that in other controls in our previous ACE studies (median survival of 21 days) (52). While the overall graft survival in the MR-1 treated mice in this study was for some reason less than what we previously observed in a similar model (52), MR-1 treatment still resulted in significant prolongation in the survival of the ACE-transplanted islet allografts compared to the control groups (**Figure 3**).

A distinctive advantage of the ACE-transplantation is that graft integrity can be monitored by direct noninvasive visualization, and longitudinal images of the same individual islets can be used to document the survival of the graft. This is illustrated in **Figures 4A, B** with two representative sets of longitudinal images showing the whole eye with pancreatic islets engrafted on top of the iris and the corresponding confocal micrographs of representative islets from an MR-1 treated mouse, which rejected its islets by day 56, and a DRI-C21041 treated mouse that did not reject its islet grafts up to the end of the follow-up on day 115 post-transplant. For the rejected islet shown, a reduction of its volume was already evident by day 35 (POD35), and the islet was no longer visible by day 56 post-transplant due to its complete immune destruction (**Figure 4A**). In contrast, the structural integrity of the islet in the tolerant DRI-C21041 treated mouse was maintained, and its volume remained unchanged until the end of the experiment (**Figure 4B**). *In vivo* volumetric analysis in confocal *z*-stacks (i.e., in 3D) of the individual islets in the DRI-C21041 and MR-1 treated mice highlighted the difference in the average change in islet volume between the two groups over time (**Figure 4C**). Further, *ex vivo* analysis in such islets (after necropsy) by immunostaining for glucagon and insulin also confirmed the sustained graft function in the DRI-C21041 treated recipients until the end of the studies on day 115 post-transplant (**Figure 4D**).

### 3.3 Suppression of immune cell infiltration

**Figure 5:**
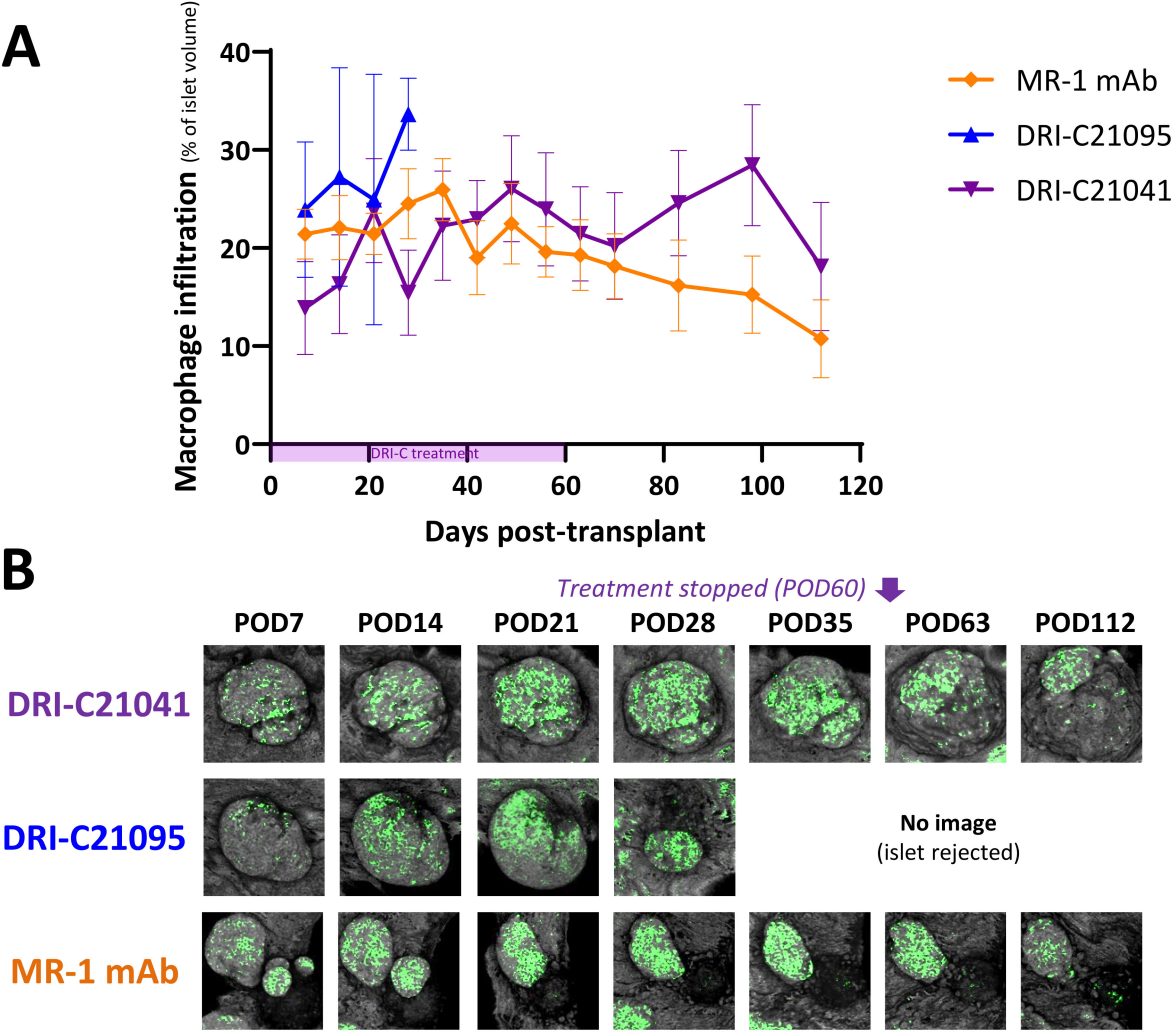
Time-course of macrophage infiltration into rejected versus tolerated islet allografts. **(A)** Longitudinal analysis of macrophage infiltration into rejected or tolerated islet allografts (*n* = 4–16) in selected representative recipient mice (*n* = 1–2) from the various treatment groups and **(B)** representative longitudinal images of rejected or tolerated single islets from selected recipients. Macrophages in these mice were visualized based on GFP expression and islets by backscatter (see Methods for details). The same islets were repeatedly imaged at the indicated time-points until rejection onset or stop of follow-up on day 112 post-transplant (POD = post-operative day). Images shown as max projection of *z*-stacks of confocal micrographs spanning the entire islets and where the infiltration analysis was performed in 3D (see Methods). Infiltration data shown as the means ± SEM of the individual islets in the imaged mice from the various treatment groups. Not all mice in each group were imaged, and data shown for DRI-C21095- and vehicle-treated as well as naïve controls correspond to mice that rejected their islet allografts.

**Figure 6:**
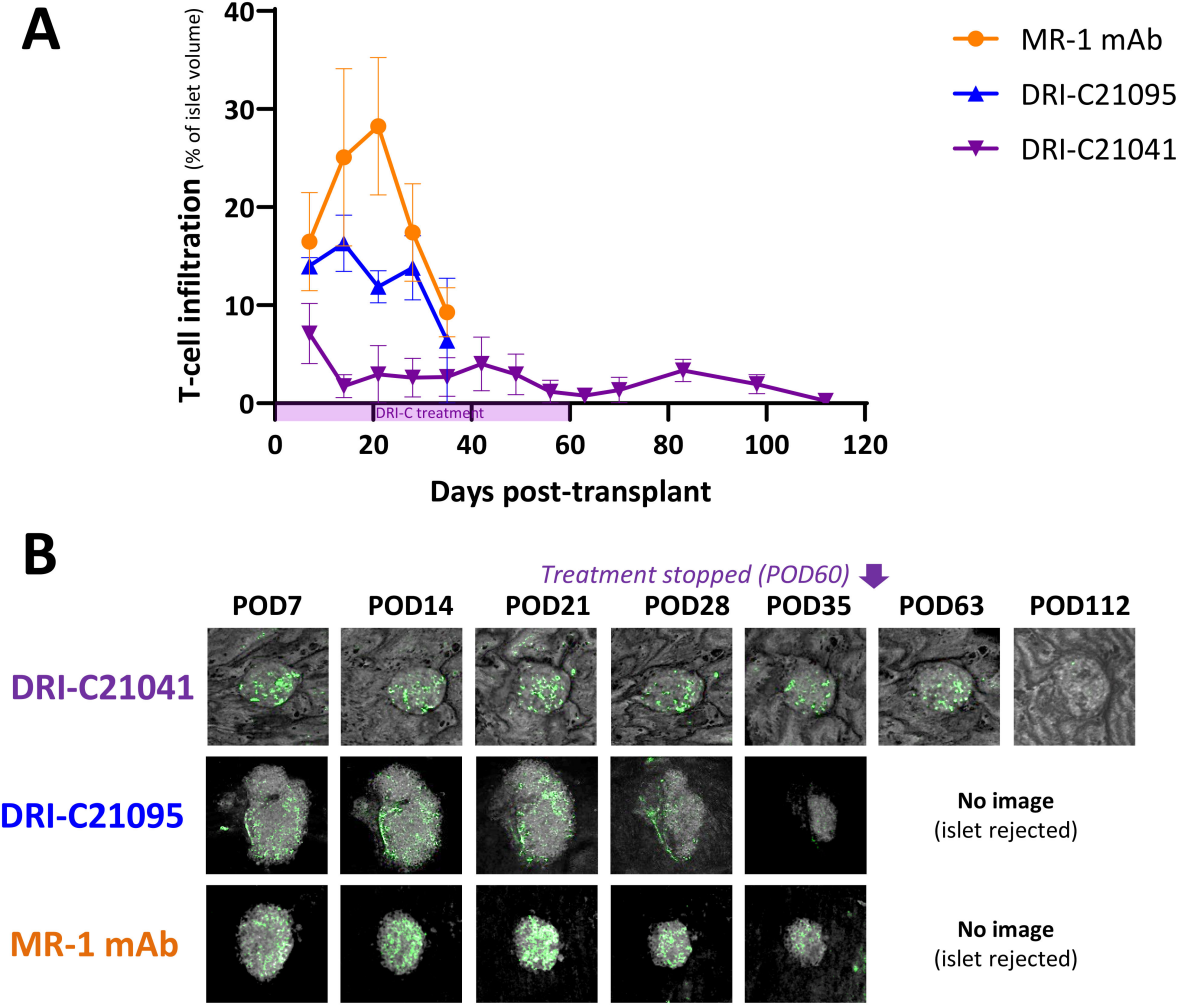
T cell infiltration kinetics into rejected versus tolerated islet allografts. **(A)** Time-course of T cell infiltration quantified in islets (*n* = 3–12) of selected representative recipient mice (*n* = 1–2) from the various treatment groups and **(B)** representative longitudinal images of single islets from selected recipients that either rejected or tolerated their ACE-transplanted islets. T cells in these mice were visualized based on GFP expression in the recipients and islets by backscatter (see Methods for details). Images (*z*-stack confocal micrographs shown as max projection) show the same islets imaged noninvasively and longitudinally at the indicated time-points until rejection onset or stop of follow-up. Infiltration data shown as the means ± SEM for each treatment group until imaging was stopped either due to islet rejection in the followed/imaged mice or at the end of follow-up of tolerant mice (POD112).

Because islets transplanted in the ACE can be followed via direct noninvasive imaging, we monitored *in situ* the graft infiltration by GFP-expressing macrophages and T cells (see Methods for details) and quantified the *in vivo* infiltration kinetics in mice that either rejected or tolerated their islet allografts. Regardless of treatment, macrophages initially infiltrated islets to similar degrees in recipients that ended up either rejecting or tolerating their allografts, but their relative abundance noticeably increased in rejected islets whose volume was progressively reduced (**Figure 5**). Additional studies in mice with GFP-expressing T cells further showed that blocking the CD40–CD40L interaction with either our SMIs or MR-1 decreased the T cell infiltration into the islet allografts. Interestingly, while the abundance of macrophages within tolerated islets remained relatively unchanged in the DRI-C21041 and MR-1 treated recipients until the end of follow-up on post-transplant day 112 (**Figure 5**), tolerant mice treated with our DRI-C compounds had markedly reduced initial T cell infiltration in the first 21 days after the transplant compared to the MR-1 treated counterparts, and this persisted until the end of the follow-up on day 112 post-transplant in DRI-C21041 treated mice (**Figure 6**).

### 3.4 Prevention of diabetes onset

**Figure 7:**
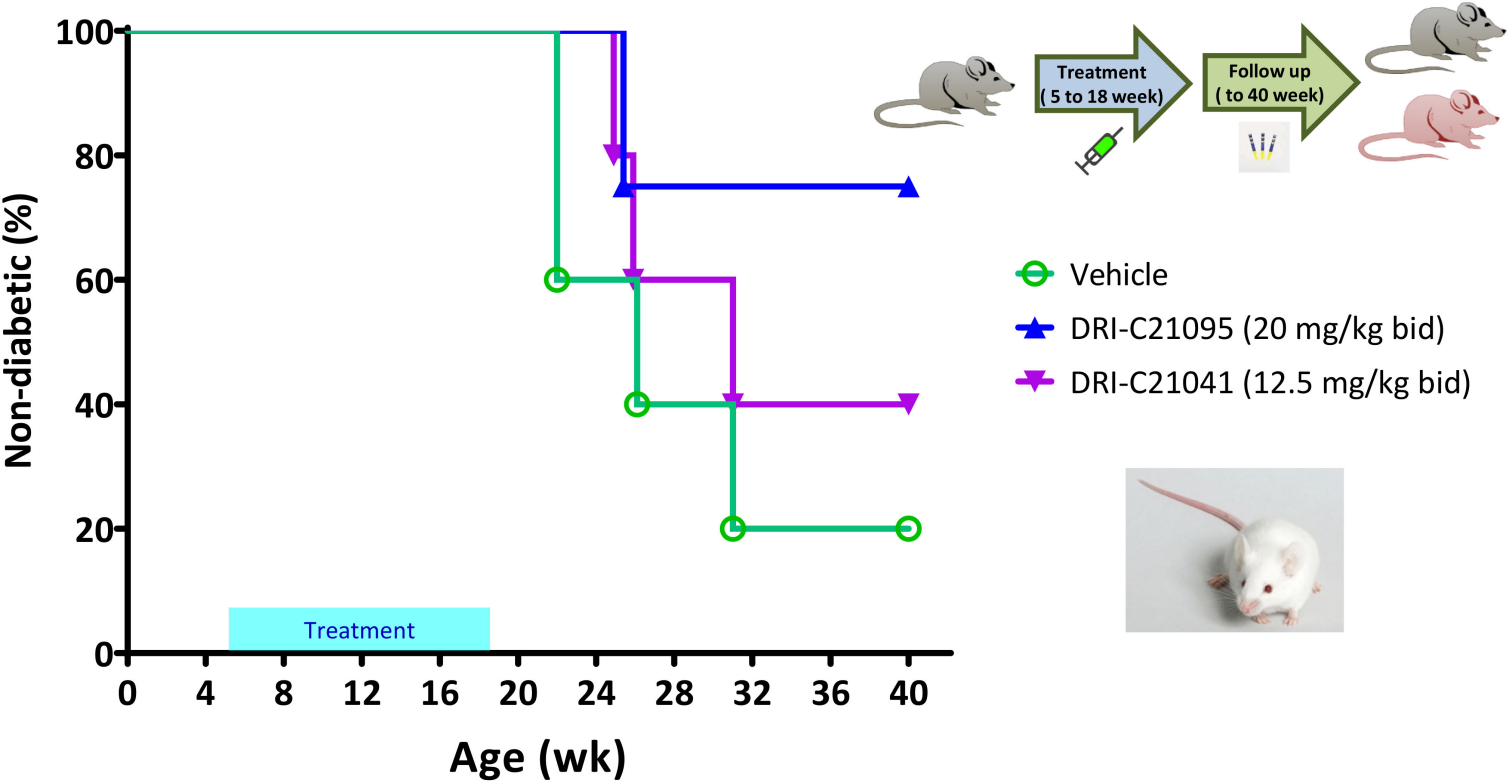
Prevention of diabetes in NOD mice by DRI-C21041 and DRI-C21095. NOD mice were treated from 5 to 18 weeks of age with DRI-C21095 and DRI-C21041 at doses of 20 and 12.5 mg/kg b.i.d., respectively. Control mice were treated with the vehicle alone during the same period. Mice were monitored for diabetes onset up to 40 weeks of age, and the percentage (%) of mice surviving diabetes-free (non-diabetic) in each treatment group is shown in a Kaplan-Meier survival plot (*n* = 4–5 mice per group). The median age of diabetes-free survival was 26 weeks in the vehicle-treated controls versus 31 weeks in those treated with DRI-C21041 and remained undetermined in the DRI-C21095–treated mice (did not cross the 50% threshold).

In addition to evaluating the immune suppressive effects of our SMIPPIs in allogeneic islet transplant models, we also assessed their ability to prevent the onset of autoimmune T1D in NOD mice in an exploratory study using female animals as customary due to their higher incidence rate of spontaneous diabetes. In light of previous publications studying CD40–CD40L inhibition in the same model (54, 60, 94), we administered treatments here from 5 to 18 weeks of age and monitored the diabetes incidence by regular glycemia measurements during an extended follow-up period until the mice reached 40 weeks of age. Results indicated that while hyperglycemia (i.e., diabetes onset) occurred in the typical 80% range in the control-treated animals, the incidence was reduced in the groups treated with our SMIs: it was reduced to 60% and 25% in the DRI-C21041- and DRI-C21095-treated mice, respectively (**Figure 7**). The median age of diabetes-free survival was 26 weeks in the vehicle-treated controls versus 31 weeks in those treated with DRI-C21041 and >40 weeks in the DRI-C21095 treated mice since they did not cross the 50% threshold (“undetermined”). We also performed exploratory immunophenotyping to identify indications of possible lasting treatment effects on immune cell subpopulations in the mice that did not become diabetic. Results can be considered as indicative only since they are from a small number of mice (one or two per group) and well after the termination of treatment (at week 40 vs 18 of age), nevertheless, they suggested possible persistent reduction in natural killer cells (NK; NK1.1^+^CD3^–^) and central memory T cells (CD4 and CD8 Tcm; CD44^+^CD62L^+^) and a concomitant increase in regulatory T cells (Treg; CD25^+^FoxP3^+^) (**Supplementary Figure 1**).

### 3.5 Pharmacokinetics

**Figure 8:**
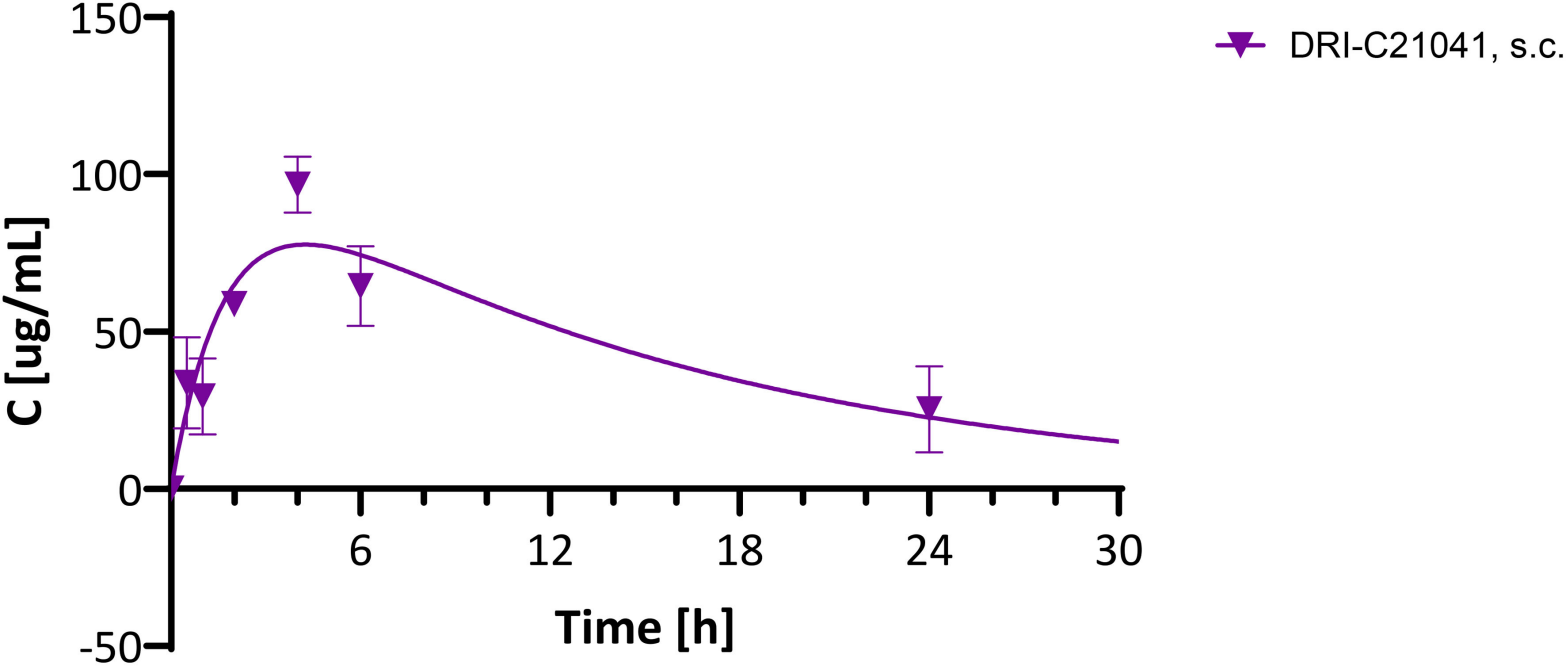
Pharmacokinetic evaluation of DRI-C21041 in mice. Concentration-time profile of DRI-C21041 in plasma following a single dose administration (30 mg/kg, s.c.) to male and female C57BL/6 mice. Experimental data (mean ± SD, *n* = 2–4 mice; purple symbols) shown fitted with a standard one compartment, first order absorption, first order elimination PK model (line).

Because DRI-C21041 performed unexpectedly well in the ACE allo-transplant model compared to the ester-containing DRI-C21095 (**Figure 1**), which has shown higher potency *in vitro* (87, 88) and performed well in the kidney allo-transplant and diabetes prevention models, we carried out a brief pharmacokinetic (PK) evaluation in C57BL/6 mice. A single dose (30 mg/kg) was administered s.c. (same route as in the studies used for efficacy assessments), and plasma concentrations were quantified in samples collected at predefined time-points after the injection (0, 0.5, 1, 2, 4, 6, and 24 h). Results indicated that DRI-C21041 has an acceptably long half-life (*t*_1/2_ = 10.8 h) (**Figure 8**), considerably better than the ester-containing DRI-C21045 that we evaluated in an earlier study (*t*_1/2_ ≈ 2 h) (87). This is not surprising as ester-containing compounds are susceptible to quick metabolic degradation by abundantly present esterases, often resulting in shorter half-lives – an effect further exacerbated in rodents as they tend to metabolize ester-containing drugs (especially aliphatic esters) much faster than humans due to their known higher esterase activity (95).

## 4 Discussion

T1D, a disease in which the β-cells of the pancreatic islets are destroyed by an autoimmune process resulting in lifelong insulin deficiency (96–99), affects more than 8 million individuals worldwide and, as prevalence increases due to improved life-expectancy, it now affects about 1 in 200 adults and children older than 10 years of age in the United States (99–102). More than a century after the introduction of treatment with insulin, T1D still represents a disease with considerable therapeutic need, not only because of the burden of constant administration of exogenous insulin, but also because chronic and degenerative complications still occur in a sizable fraction of T1D patients despite considerable improvements in diabetes management and care (103–105). There is still excess mortality and loss of 10–20 life-years among those diagnosed with T1D even in high-income countries, and a much higher loss, >40 life-years, in low-income countries (101, 106, 107). Compared to the general population, all-cause mortality risk is about three-fold higher in people with T1D (108). Alarmingly, the incidence of T1D is increasing worldwide at a rate of 2–5% per year (97, 109–111), a phenomenon that seems to be occurring with other autoimmune diseases as well (112). Notably, despite many clinical trials of various immune interventions to prevent its onset, T1D remains the only autoimmune condition without a truly effective immunotherapy. Even the most successful immune interventions explored so far (e.g., cyclosporine, otelixizumab, rituximab, and abatacept) have achieved only a few months delay in the autoimmune-driven decline of insulin production (113–115). Teplizumab, which was recently approved by the FDA as the very first disease-modifying intervention for T1D (116), has shown a somewhat more promising delay in T1D onset (median of about 2 years) (117, 118), but it is restricted to select high-risk patients. Therefore, the need for effective immunotherapies for T1D remains critical, and controlling the associated autoimmune process will likely require novel immunomodulatory approaches (119). As ICP targets show particular promise (62), SMIPPIs targeting immune costimulation, such as those discussed here (i.e., CD40–CD40L), could ultimately lead to effective immunomodulatory therapeutics for the prevention of T1D and possibly other autoimmune diseases as well.

Furthermore, improved immunosuppressive therapies are also desperately needed for organ- and cell-transplant recipients to reduce or eliminate the serious side effects associated with current therapies that require chronic administration to avoid rejection and maintain the graft function. Costimulatory blockade is particularly promising in this aspect as well because it can be antigen/activation-specific and thus, less broadly immunosuppressive than current therapies and can lead to tolerance by allowing antigen recognition in the absence of costimulation (52, 63, 120–122). Because islets and especially β-cells are sensitive to commonly used immunosuppressive drugs (123, 124), there is an even more pronounced need for refined immunosuppressive strategies in β-cell replacement therapies, which can restore metabolic control more efficiently than exogeneous insulin therapy and prevent the serious long-term complications and co-morbidities associated with T1D (125–128). This is especially important now that (*i*) the FDA approved islet transplantation as a cell transplant therapy for the treatment of T1D (129) and (*ii*) stem cell therapies, which can provide renewable sources of insulin producing cells and promote the applicability of transplant therapy on a much wider scale, are beginning to show promise in T1D and have reached the clinical development phase (130–133).

In our previous work, we identified novel DRI-C compounds that inhibited the CD40–CD40L PPI with high nanomolar to low micromolar potency (IC_50_) and with >30-fold selectivity versus other TNF superfamily PPIs, namely OX40–OX40L, BAFFR–BAFF, and TNF-R1–TNFα (87, 88). Protein thermal shift analysis suggested that these SMIPPIs bind CD40L rather than CD40. The lead compounds used here, DRI-C21095 and DRI-C21041, inhibited CD40–CD40L binding in our cell-free ELISA assay with IC_50_ values of 19 and 87 nM, respectively (88). Their activities were also confirmed in cell-based assays. For example, DRI-C21041 inhibited the CD40L-induced activation of NF-κB biosensor cells with an IC_50_ of 10.3 μM and that of primary human B lymphocytes with an IC_50_ of 13.2 μM (87). DRI-C21095 had an IC_50_ of 6.0 μM in the NF-κB biosensor cell assay (88). *In vivo* activity was also confirmed previously in the short-term assay of alloantigen-induced T cell expansion in a draining lymph node (87, 88). Here, we confirmed the *in vivo* efficacy of our lead SMIs in longer-term transplantation- and autoimmune disease-relevant mouse models: two full MHC-mismatched allogeneic islet transplantation models (in the kidney subcapsular space and the anterior chamber of the eye - ACE, respectively) and a T1D prevention model in the diabetes-prone NOD mice.

The studies with islet allografts under the kidney capsule confirmed the immunosuppressive effects of both SMIs in a dose-dependent manner. At their highest doses tested here, both DRI-C21041 (12.5 mg/kg b.i.d.) and DRI-C21095 (20 mg/kg b.i.d.) significantly prolonged the survival and function of islet allografts in ~50% of recipients long after terminating the treatments (**Figure 2**) at doses that are roughly equivalent with that of the MR-1 antibody used as positive control (250 μg/mouse ≈ 10 mg/kg) considering the differences in molecular weights, potencies (IC_50_), and elimination half-lives (*t*_1/2_). Similarly, studies in the ACE-platform confirmed the long-term survival of islet allografts in recipients treated with either compound, and 80% of the DRI-C21041 treated recipients maintained structurally intact islets for >50 days after stopping treatment (**Figure 3**). We further exploited the ability of the ACE-platform that allows the detailed 3D spatial mapping of cellular level allo- and auto-immune reactions in and around the grafts and the longitudinal *in situ* tracking of infiltrating immune cells to derive kinetic profiles characteristic of specific cell subtypes. These studies revealed that treatment with the DRI-C compounds markedly reduced the infiltration of the allografts by T effector cells (**Figure 6**). Given the overwhelming evidence that allo- and auto-immune responses against ACE-transplanted islets mirror those in the pancreas and other transplant sites (92, 134–140), the current findings suggest that our SMIPPIs likely inhibited the activation and priming of T effector cells, which in turn reduced their infiltration into the islet allografts and consequently promoted the long-term survival and function of the islets in both transplant sites (i.e., kidney and ACE) and, possibly, the native pancreas of the NOD mice that never progressed to diabetes (**Figure 7**). Our exploratory immunophenotyping study in splenocytes also indicated a trend towards reduced central memory T cells among splenocytes and a concomitant increase in Tregs in association with DRI-C21041 and DRI-C21095 treatments in NOD mice (**Supplementary Figure 1**), further suggestive of peripheral immune regulation. These results are in overall agreement with effects expected from blockade of this important costimulatory interaction between CD40 and CD40L, which is mainly expressed on activated CD4^+^ T cells, known to play important roles in the promotion of germinal center formation and the production of class-switched antibodies as well as in the triggering of pro-inflammatory responses (34). Hence, the CD40–CD40L ICP represents an important therapeutic target in transplant therapy (33) and autoimmune diseases (34) as demonstrated by the large number of novel biologics being developed against it including those mentioned in the Introduction, such as letolizumab, dapirolizumab pegol, frexalimab, tegoprubart, TNX-1500, dazodalibep, or iscalimab.

The NOD mouse, an inbred strain developed from a line originally intended as a cataract-prone mouse strain (141), is by far the most commonly used animal model for autoimmune T1D (142–147). NOD mice develop diabetes spontaneously (typically, in about 60%–90% of females and 20%–40% of males between the age of 12 to 30 weeks) reproducing many aspects of the human autoimmune disease (e.g., presence of islet-specific autoantibodies, inflammation of pancreatic islets, and dependence on MHC alleles), but also with some important differences such as more severe insulitis and gender bias. An important role for the CD40–CD40L axis in T1D development has been already shown (55, 57): for example, diabetes did not develop in CD40L^–/–^ NOD mice (56) and treatment with anti-CD40L mAb (54, 94) or CD40 peptide-inhibitors (60) abrogated the disease in regular NOD mice if treatment was initiated early enough. Our results in the NOD model are promising as they showed considerable reduction in the rate of onset (**Figure 7**); however, they must be treated with caution since, on one hand, they were obtained on a relatively small number of mice and, on the other, results in NODs in general do not translate well to humans. More than one hundred different successful preventive interventions in NOD mice have been reported already while their therapeutic success in humans has remained limited (143, 145). Nevertheless, the current findings showing the effect of our SMIPPIs in preventing T1D onset in NOD mice support their efficacy and further development for potential clinical application. DRI-C21095 seemed particularly effective in the NOD T1D onset prevention model (**Figure 7**) whereas DRI-C21041 in the ACE islet allotransplant model (**Figure 3**); future studies will explore the potential relevance of this for different clinical applications including in diabetic transplant recipients where both the allo- and autoimmune responses are present.

## 5 Conclusion

Results here confirm the *in vivo* therapeutic potential of DRI-C21041 and DRI-C21095, our new SMIs of the CD40–CD40L costimulatory PPI, as novel immune modulatory agents. Both agents showed considerable promise in two allogeneic islet transplant models, even suggesting the possibility of inducing operational immune tolerance, as well as in preventing autoimmune diabetes in the NOD mouse model. DRI-C21041 showed better *in vivo* efficacy than anticipated, especially in the ACE allotransplant model, likely due to its longer elimination half-life, which should allow for once daily administration in future studies. In summary, the current findings not only demonstrate the long-term *in vivo* efficacy of our new SMIs, but they also (*i*) provide further evidence that the CD40–CD40L PPI is susceptible to small-molecule inhibition and (*ii*) reinforce the potential for the induction of operational transplant immune tolerance and the prevention of T1D onset through the inhibition of this costimulatory ICP. Thus, further investigation of SMIs of the CD40–CD40L PPI is warranted, as they could ultimately lead to novel alternative immunomodulatory therapeutics that are safer, less immunogenic, more controllable (e.g., have shorter half-lives), and more patient-friendly (by being easier to administer – possibly through the oral route) than protein-based biologics.

## Data Availability

The raw data supporting the conclusions of this article will be made available by the authors, without undue reservation.

## References

[1] Chen L Flies DB. Molecular mechanisms of T cell co-stimulation and co-inhibition. Nat Rev Immunol. (2013) 13:227–42. doi: 10.1038/nri3405 PMC3786574 23470321

[2] Gaikwad S Agrawal MY Kaushik I Ramachandran S Srivastava SK. Immune checkpoint proteins: Signaling mechanisms and molecular interactions in cancer immunotherapy. Semin Cancer Biol. (2022) 86:137–50. doi: 10.1016/j.semcancer.2022.03.014 35341913

[3] Huang C Zhu HX Yao Y Bian ZH Zheng YJ Li L. Immune checkpoint molecules. Possible future therapeutic implications in autoimmune diseases. J Autoimmun. (2019) 104:102333. doi: 10.1016/j.jaut.2019.102333 31564474

[4] Couzin-Frankel J. Breakthrough of the year 2013. Cancer immunotherapy. Science. (2013) 342:1432–3. doi: 10.1126/science.342.6165.1432 24357284

[5] Hoos A. Development of immuno-oncology drugs - from CTLA4 to PD1 to the next generations. Nat Rev Drug Discovery. (2016) 15:235–47. doi: 10.1038/nrd.2015.35 26965203

[6] Leader B Baca QJ Golan DE. Protein therapeutics: a summary and pharmacological classification. Nat Rev Drug Discovery. (2008) 7:21–39. doi: 10.1038/nrd2399 18097458

[7] Ryman JT Meibohm B. Pharmacokinetics of monoclonal antibodies. CPT Pharmacometrics Syst Pharmacol. (2017) 6:576–88. doi: 10.1002/psp4.12224 PMC5613179 28653357

[8] Huck BR Kötzner L Urbahns K. Small molecules drive big improvements in immuno-oncology therapies. Angew Chem Int Ed Engl. (2018) 57:4412–28. doi: 10.1002/anie.201707816 PMC5900885 28971564

[9] Downing NS Shah ND Aminawung JA Pease AM Zeitoun JD Krumholz HM. Postmarket safety events among novel therapeutics approved by the US Food and Drug Administration between 2001 and 2010. J Am Med Assoc (JAMA). (2017) 317:1854–63. doi: 10.1001/jama.2017.5150 PMC5815036 28492899

[10] Sathish JG Sethu S Bielsky MC de Haan L French NS Govindappa K. Challenges and approaches for the development of safer immunomodulatory biologics. Nat Rev Drug Discovery. (2013) 12:306–24. doi: 10.1038/nrd3974 PMC7097261 23535934

[11] Martins F Sofiya L Sykiotis GP Lamine F Maillard M Fraga M. Adverse effects of immune-checkpoint inhibitors: epidemiology, management and surveillance. Nat Rev Clin Oncol. (2019) 16:563–80. doi: 10.1038/s41571-019-0218-0 31092901

[12] Giannoukakis N Phillips B Trucco M. Toward a cure for type 1 diabetes mellitus: diabetes-suppressive dendritic cells and beyond. Pediatr Diabetes. (2008) 9:4–13. doi: 10.1111/j.1399-5448.2008.00401.x 18540865

[13] Cochrane GM Horne R Chanez P. Compliance in asthma. Respir Med. (1999) 93:763–9. doi: 10.1016/s0954-6111(99)90260-3 10603624

[14] Moia M Mantovani LG Carpenedo M Scalone L Monzini MS Cesana G. Patient preferences and willingness to pay for different options of anticoagulant therapy. Intern Emerg Med. (2013) 8:237–43. doi: 10.1007/s11739-012-0844-3 22926743

[15] Arkin MR Wells JA. Small-molecule inhibitors of protein-protein interactions: progressing towards the dream. Nat Rev Drug Discovery. (2004) 3:301–17. doi: 10.1038/nrd1343 15060526

[16] Wells JA McClendon CL. Reaching for high-hanging fruit in drug discovery at protein-protein interfaces. Nature. (2007) 450:1001–9. doi: 10.1038/nature06526 18075579

[17] Buchwald P. Small-molecule protein-protein interaction inhibitors: therapeutic potential in light of molecular size, chemical space, and ligand binding efficiency considerations. IUBMB Life. (2010) 62:724–31. doi: 10.1002/iub.383 20979208

[18] Milroy LG Grossmann TN Hennig S Brunsveld L Ottmann C. Modulators of protein-protein interactions. Chem Rev. (2014) 114:4695–748. doi: 10.1021/cr400698c 24735440

[19] Scott DE Bayly AR Abell C Skidmore J. Small molecules, big targets: drug discovery faces the protein-protein interaction challenge. Nat Rev Drug Discovery. (2016) 15:533–50. doi: 10.1038/nrd.2016.29 27050677

[20] Bojadzic D Buchwald P. Toward small-molecule inhibition of protein-protein interactions: General aspects and recent progress in targeting costimulatory and coinhibitory (immune checkpoint) interactions. Curr Top Med Chem. (2018) 18:674–99. doi: 10.2174/1568026618666180531092503 PMC6067980 29848279

[21] Buchwald P. Developing small-molecule inhibitors of protein-protein interactions involved in viral entry as potential antivirals for COVID-19. Front Drug Discovery. (2022) 2:2022.898035. doi: 10.3389/fddsv.2022.898035

[22] Wu Y Yang Z Cheng K Bi H Chen J. Small molecule-based immunomodulators for cancer therapy. Acta Pharm Sin B. (2022) 12:4287–308. doi: 10.1016/j.apsb.2022.11.007 PMC9764074 36562003

[23] Souers AJ Leverson JD Boghaert ER Ackler SL Catron ND Chen J. ABT-199, a potent and selective BCL-2 inhibitor, achieves antitumor activity while sparing platelets. Nat Med. (2013) 19:202–8. doi: 10.1038/nm.3048 23291630

[24] Gadek TR Burdick DJ McDowell RS Stanley MS Marsters JC Jr. Paris KJ. Generation of an LFA-1 antagonist by the transfer of the ICAM-1 immunoregulatory epitope to a small molecule. Science. (2002) 295:1086–9. doi: 10.1126/science.295.5557.1086 11834839

[25] Meanwell NA Krystal MR Nowicka-Sans B Langley DR Conlon DA Eastgate MD. Inhibitors of HIV-1 attachment: the discovery and development of temsavir and its prodrug fostemsavir. J Med Chem. (2018) 61:62–80. doi: 10.1021/acs.jmedchem.7b01337 29271653

[26] Kesselheim AS Avorn J. The most transformative drugs of the past 25 years: a survey of physicians. Nat Rev Drug Discovery. (2013) 12:425–31. doi: 10.1038/nrd3977 23681007

[27] Vugler A O'Connell J Nguyen MA Weitz D Leeuw T Hickford E. An orally available small molecule that targets soluble TNF to deliver anti-TNF biologic-like efficacy in rheumatoid arthritis. Front Pharmacol. (2022) 13:2022.1037983. doi: 10.3389/fphar.2022.1037983 PMC9709720 36467083

[28] Chédotal H Narayanan D Povlsen K Gotfredsen CH Brambilla R Gajhede M. Small-molecule modulators of tumor necrosis factor signaling. Drug Discovery Today. (2023) 28:103575. doi: 10.1016/j.drudis.2023.103575 37003513

[29] Elgueta R Benson MJ de Vries VC Wasiuk A Guo Y Noelle RJ. Molecular mechanism and function of CD40/CD40L engagement in the immune system. Immunol Rev. (2009) 229:152–72. doi: 10.1111/j.1600-065x.2009.00782.x PMC3826168 19426221

[30] Burkly LC. CD40 pathway blockade as an approach to immunotherapy. Adv Exp Med Biol. (2001) 489:135–52. doi: 10.1007/978-1-4615-1277-6_12 11554588

[31] Quezada SA Jarvinen LZ Lind EF Noelle RJ. CD40/CD154 interactions at the interface of tolerance and immunity. Annu Rev Immunol. (2004) 22:307–28. doi: 10.1146/annurev.immunol.22.012703.104533 15032580

[32] Daoussis D Andonopoulos AP Liossis SN. Targeting CD40L: a promising therapeutic approach. Clin Diagn Lab Immunol. (2004) 11:635–41. doi: 10.1128/CDLI.11.4.635-641.2004 PMC440614 15242934

[33] Pinelli DF Ford ML. Novel insights into anti-CD40/CD154 immunotherapy in transplant tolerance. Immunotherapy. (2015) 7:399–410. doi: 10.2217/imt.15.1 25917630 PMC5441999

[34] Karnell JL Rieder SA Ettinger R Kolbeck R. Targeting the CD40-CD40L pathway in autoimmune diseases: Humoral immunity and beyond. Adv Drug Delivery Rev. (2019) 141:92–103. doi: 10.1016/j.addr.2018.12.005 30552917

[35] Parker DC Greiner DL Phillips NE Appel MC Steele AW Durie FH. Survival of mouse pancreatic islet allografts in recipients treated with allogeneic small lymphocytes and antibody to CD40 ligand. Proc Natl Acad Sci USA. (1995) 92:9560–4. doi: 10.1073/pnas.92.21.9560 PMC40841 7568172

[36] Zheng XX Markees TG Hancock WW Li Y Greiner DL Li XC. CTLA4 signals are required to optimally induce allograft tolerance with combined donor-specific transfusion and anti-CD154 monoclonal antibody treatment. J Immunol. (1999) 162:4983–90. doi: 10.4049/jimmunol.162.8.4983 10202046

[37] Molano RD Berney T Li H Cattan P Pileggi A Vizzardelli C. Prolonged islet graft survival in NOD mice by blockade of the CD40-CD154 pathway of T-cell costimulation. Diabetes. (2001) 50:270–6. doi: 10.2337/diabetes.50.2.270 11272136

[38] Molano RD Berney T Pileggi A Ricordi C Burkly L Rothstein D. Prolonged survival of allogeneic islet grafts in NOD mice treated with a combination of anti-CD45RB and anti-CD154 antibodies. Transplant Proc. (2001) 33:248–9. doi: 10.1016/s0041-1345(00)01998-9 11266802

[39] Berney T Pileggi A Molano RD Poggioli R Zahr E Ricordi C. The effect of simultaneous CD154 and LFA-1 blockade on the survival of allogeneic islet grafts in nonobese diabetic mice. Transplantation. (2003) 76:1669–74. doi: 10.1097/01.tp.0000092525.17025.d0 14688513

[40] Molano RD Pileggi A Berney T Poggioli R Zahr E Oliver R. Prolonged islet allograft survival in diabetic NOD mice by targeting CD45RB and CD154. Diabetes. (2003) 52:957–64. doi: 10.2337/diabetes.52.4.957 12663467

[41] Inverardi L Linetsky E Pileggi A Molano RD Serafini A Paganelli G. Targeted bone marrow radioablation with ^153^Samarium-lexidronam promotes allogeneic hematopoietic chimerism and donor-specific immunologic hyporesponsiveness. Transplantation. (2004) 77:647–55. doi: 10.1097/01.TP.0000112436.26473.A2 15021823

[42] Masaki H Appel MC Leahy L Leif J Paquin L Shultz LD. Anti-mouse CD154 antibody treatment facilitates generation of mixed xenogeneic rat hematopoietic chimerism, prevents wasting disease and prolongs xenograft survival in mice. Xenotransplantation. (2006) 13:224–32. doi: 10.1111/j.1399-3089.2006.00290.x 16756565

[43] Mai G Bucher P Morel P Mei J Bosco D Andres A. Anti-CD154 mAb treatment but not recipient CD154 deficiency leads to long-term survival of xenogeneic islet grafts. Am J Transplant. (2005) 5:1021–31. doi: 10.1111/j.1399-3089.2007.00402.x 15816882

[44] Mai G del Rio ML Tian J Ramirez P Buhler L Rodriguez-Barbosa JI. Blockade of the PD-1/PD-1L pathway reverses the protective effect of anti-CD40L therapy in a rat to mouse concordant islet xenotransplantation model. Xenotransplantation. (2007) 14:243–8. doi: 10.1111/j.1399-3089.2007.00402.x 17489865

[45] Jung DY Kim EY Joo SY Park JB Moon C Kim SH. Prolonged survival of islet allografts in mice treated with rosmarinic acid and anti-CD154 antibody. Exp Mol Med. (2008) 40:1–10. doi: 10.3858/emm.2008.40.1.1 18305392 PMC2679315

[46] Kenyon NS Fernandez LA Lehmann R Masetti M Ranuncoli A Chatzipetrou M. Long-term survival and function of intrahepatic islet allografts in baboons treated with humanized anti-CD154. Diabetes. (1999) 48:1473–81. doi: 10.2337/diabetes.48.7.1473 10389857

[47] Kenyon NS Chatzipetrou M Masetti M Ranuncoli A Oliveira M Wagner JL. Long-term survival and function of intrahepatic islet allografts in rhesus monkeys treated with humanized anti-CD154. Proc Natl Acad Sci USA. (1999) 96:8132–7. doi: 10.1073/pnas.96.14.8132 PMC22200 10393960

[48] Koulmanda M Smith RN Qipo A Weir G Auchincloss H Strom TB. Prolonged survival of allogeneic islets in cynomolgus monkeys after short-term anti-CD154-based therapy: nonimmunologic graft failure? Am J Transplant. (2006) 6:687–96. doi: 10.1111/j.1600-6143.2006.01235.x PMC3779922 16539625

[49] Cardona K Korbutt GS Milas Z Lyon J Cano J Jiang W. Long-term survival of neonatal porcine islets in nonhuman primates by targeting costimulation pathways. Nat Med. (2006) 12:304–6. doi: 10.1038/nm1375 16501570

[50] van der Windt DJ Bottino R Casu A Campanile N Smetanka C He J. Long-term controlled normoglycemia in diabetic non-human primates after transplantation with hCD46 transgenic porcine islets. Am J Transplant. (2009) 9:2716–26. doi: 10.1111/j.1600-6143.2009.02850.x 19845582

[51] Bottino R Knoll MF Graeme-Wilson J Klein EC Ayares D Trucco M. Safe use of anti-CD154 monoclonal antibody in pig islet xenotransplantation in monkeys. Xenotransplantation. (2017) 24:e12283. doi: 10.1111/xen.12283 PMC5332295 28058735

[52] Abdulreda MH Berman DM Shishido A Martin C Hossameldin M Tschiggfrie A. Operational immune tolerance towards transplanted allogeneic pancreatic islets in mice and a non-human primate. Diabetologia. (2019) 62:811–21. doi: 10.1007/s00125-019-4814-4 PMC6451664 30701283

[53] Peters AL Stunz LL Bishop GA. CD40 and autoimmunity: The dark side of a great activator. Semin Immunol. (2009) 21:293–300. doi: 10.1016/j.smim.2009.05.012 19595612 PMC2753170

[54] Balasa B Krahl T Patstone G Lee J Tisch R McDevitt HO. CD40 ligand-CD40 interactions are necessary for the initiation of insulitis and diabetes in nonobese diabetic mice. J Immunol. (1997) 159:4620–7. doi: 10.4049/jimmunol.159.9.4620 9379064

[55] Homann D Jahreis A Wolfe T Hughes A Coon B van Stipdonk MJ. CD40L blockade prevents autoimmune diabetes by induction of bitypic NK/DC regulatory cells. Immunity. (2002) 16:403–15. doi: 10.1016/s1074-7613(02)00290-x 11911825

[56] Bour-Jordan H Salomon BL Thompson HL Szot GL Bernhard MR Bluestone JA. Costimulation controls diabetes by altering the balance of pathogenic and regulatory T cells. J Clin Invest. (2004) 114:979–87. doi: 10.1172/JCI200420483 PMC518661 15467837

[57] Baker RL Wagner DH Jr. Haskins K. CD40 on NOD CD4 T cells contributes to their activation and pathogenicity. J Autoimmun. (2008) 31:385–92. doi: 10.1016/j.jaut.2008.09.001 18951762

[58] Wagner DH Jr. Vaitaitis G Sanderson R Poulin M Dobbs C Haskins K. Expression of CD40 identifies a unique pathogenic T cell population in type 1 diabetes. Proc Natl Acad Sci USA. (2002) 99:3782–7. doi: 10.1073/pnas.052247099 PMC122601 11891296

[59] Baker RL Mallevaey T Gapin L Haskins K. T cells interact with T cells via CD40-CD154 to promote autoimmunity in type 1 diabetes. Eur J Immunol. (2012) 42:672–80. doi: 10.1002/eji.201142071 PMC3697870 22488364

[60] Vaitaitis GM Olmstead MH Waid DM Carter JR. Wagner DH, Jr. A CD40-targeted peptide controls and reverses type 1 diabetes in NOD mice. Diabetologia. (2014) 57:2366–73. doi: 10.1007/s00125-014-3342-5 PMC4183717 25104468

[61] Vaitaitis GM Waid DM Yussman MG Wagner DH Jr. CD40-mediated signalling influences trafficking, T-cell receptor expression, and T-cell pathogenesis, in the NOD model of type 1 diabetes. Immunology. (2017) 152:243–54. doi: 10.1111/imm.12761 PMC5588813 28542921

[62] Spicer P Runkel L. Costimulatory pathway targets for autoimmune and inflammatory conditions: clinical successes, failures, and hope for the future. Expert Opin Investig Drugs. (2019) 28:99–106. doi: 10.1080/13543784.2019.1557146 30661473

[63] Croft M Benedict CA Ware CF. Clinical targeting of the TNF and TNFR superfamilies. Nat Rev Drug Discovery. (2013) 12:147–68. doi: 10.1038/nrd3930 PMC3625401 23334208

[64] Song Y Buchwald P. TNF superfamily protein-protein interactions: feasibility of small-molecule modulation. Curr Drug Targets. (2015) 16:393–408. doi: 10.2174/1389450116666150223115628 25706111 PMC4408546

[65] Kawai T Andrews D Colvin RB Sachs DH Cosimi AB. Thromboembolic complications after treatment with monoclonal antibody against CD40 ligand. Nat Med. (2000) 6:114. doi: 10.1038/72162 10655073

[66] Boumpas DT Furie R Manzi S Illei GG Wallace DJ Balow JE. A short course of BG9588 (anti-CD40 ligand antibody) improves serologic activity and decreases hematuria in patients with proliferative lupus glomerulonephritis. Arthritis Rheum. (2003) 48:719–27. doi: 10.1002/art.10856 12632425

[67] Koyama I Kawai T Andrews D Boskovic S Nadazdin O Wee SL. Thrombophilia associated with anti-CD154 monoclonal antibody treatment and its prophylaxis in nonhuman primates. Transplantation. (2004) 77:460–2. doi: 10.1097/01.tp.0000110291.29370.c0 14966427

[68] Roth GA Zuckermann A Klepetko W Wolner E Ankersmit HJ Moser B. Thrombophilia associated with anti-CD154 monoclonal antibody treatment and its prophylaxis in nonhuman primates. Transplantation. (2004) 78:1238–9. doi: 10.1097/01.tp.0000135457.69220.5b 15502729

[69] Couzin J. Drug discovery. Magnificent obsession. Sci. (2005) 307:1712–5. doi: 10.1126/science.307.5716.1712 15774740

[70] Mirabet M Barrabes JA Quiroga A Garcia-Dorado D. Platelet pro-aggregatory effects of CD40L monoclonal antibody. Mol Immunol. (2008) 45:937–44. doi: 10.1016/j.molimm.2007.08.006 17959249

[71] Ramanujam M Steffgen J Visvanathan S Mohan C Fine JS Putterman C. Phoenix from the flames: Rediscovering the role of the CD40-CD40L pathway in systemic lupus erythematosus and lupus nephritis. Autoimmun Rev. (2020) 19:102668. doi: 10.1016/j.autrev.2020.102668 32942031

[72] Xie JH Yamniuk AP Borowski V Kuhn R Susulic V Rex-Rabe S. Engineering of a novel anti-CD40L domain antibody for treatment of autoimmune diseases. J Immunol. (2014) 192:4083–92. doi: 10.4049/jimmunol.1303239 24670803

[73] Kim SC Wakwe W Higginbotham LB Mathews DV Breeden CP Stephenson AC. Fc-Silent anti-CD154 domain antibody effectively prevents nonhuman primate renal allograft rejection. Am J Transplant. (2017) 17:1182–92. doi: 10.1111/ajt.14197 PMC5409881 28097811

[74] Tang T Cheng X Truong B Sun L Yang X Wang H. Molecular basis and therapeutic implications of CD40/CD40L immune checkpoint. Pharmacol Ther. (2021) 219:107709. doi: 10.1016/j.pharmthera.2020.107709 33091428 PMC7886970

[75] Tocoian A Buchan P Kirby H Soranson J Zamacona M Walley R. First-in-human trial of the safety, pharmacokinetics and immunogenicity of a PEGylated anti-CD40L antibody fragment (CDP7657) in healthy individuals and patients with systemic lupus erythematosus. Lupus. (2015) 24:1045–56. doi: 10.1177/0961203315574558 25784719

[76] Furie RA Bruce IN Dorner T Leon MG Leszczynski P Urowitz M. Phase 2, randomized, placebo-controlled trial of dapirolizumab pegol in patients with moderate-to-severe active systemic lupus erythematosus. Rheumatology. (2021) 60:5397–407. doi: 10.1093/rheumatology/keab381 PMC9194804 33956056

[77] Vermersch P Granziera C Mao-Draayer Y Cutter G Kalbus O Staikov I. Inhibition of CD40L with frexalimab in multiple sclerosis. N Engl J Med. (2024) 390:589–600. doi: 10.1056/nejmoa2309439 38354138

[78] Perrin S Gill A Gill C Vieira F Thompson K Lincecum J. The development and characterization of AT1501, an anti CD40L antibody lacking Fc effector function. Am J Transplant. (2021) 21:Abstr. 512. https://atcmeetingabstracts.com/abstract/the-development-and-characterization-of-at1501-an-anti-cd40l-antibody-lacking-fc-effector-function/.

[79] Miura S Abady Z Pollok F Ma M Kinoshita K Fogarty S. TNX-1500, an Fc-modified anti-CD154 antibody, prolongs nonhuman primate cardiac allograft survival. J Heart Lung Transplant. (2022) 41:S148. doi: 10.1016/j.healun.2022.01.348 PMC10524282 37030662

[80] Karnell JL Albulescu M Drabic S Wang L Moate R Baca M. A CD40L-targeting protein reduces autoantibodies and improves disease activity in patients with autoimmunity. Sci Trans Med. (2019) 11:eaar6584. doi: 10.1126/scitranslmed.aar6584 31019027

[81] Singh AK Goerlich CE Zhang T Lewis BGT Hershfeld A Mohiuddin MM. CD40-CD40L blockade: update on novel investigational therapeutics for transplantation. Transplantation. (2023) 107:1472–81. doi: 10.1097/tp.0000000000004469 PMC10287837 36584382

[82] St Clair EW Baer AN Ng WF Noaiseh G Baldini C Tarrant TK. CD40 ligand antagonist dazodalibep in Sjögren's disease: a randomized, double-blinded, placebo-controlled, phase 2 trial. Nat Med. (2024) 30:1583–92. doi: 10.1038/s41591-024-03009-3 PMC11186761 38839899

[83] Anwar IJ Berman DM DeLaura I Gao Q Willman MA Miller A. The anti-CD40L monoclonal antibody AT-1501 promotes islet and kidney allograft survival and function in nonhuman primates. Sci Trans Med. (2023) 15:eadf6376. doi: 10.1126/scitranslmed.adf6376 PMC10990482 37647390

[84] Fisher BA Mariette X Papas A Grader-Beck T Bootsma H Ng WF. Safety and efficacy of subcutaneous iscalimab (CFZ533) in two distinct populations of patients with Sjögren's disease (TWINSS): week 24 results of a randomised, double-blind, placebo-controlled, phase 2b dose-ranging study. Lancet. (2024) 404:540–53. doi: 10.1016/s0140-6736(24)01211-x 39096929

[85] Margolles-Clark E Jacques-Silva MC Ganesan L Umland O Kenyon NS Ricordi C. Suramin inhibits the CD40–CD154 costimulatory interaction: a possible mechanism for immunosuppressive effects. Biochem Pharmacol. (2009) 77:1236–45. doi: 10.1016/j.bcp.2009.01.001 19283894

[86] Margolles-Clark E Umland O Kenyon NS Ricordi C Buchwald P. Small molecule costimulatory blockade: organic dye inhibitors of the CD40-CD154 interaction. J Mol Med. (2009) 87:1133–43. doi: 10.1007/s00109-009-0519-3 19707732

[87] Chen J Song Y Bojadzic D Tamayo-Garcia A Landin AM Blomberg BB. Small-molecule inhibitors of the CD40-CD40L costimulatory protein-protein interaction. J Med Chem. (2017) 60:8906–22. doi: 10.1021/acs.jmedchem.7b01154 PMC5823691 29024591

[88] Bojadzic D Chen J Alcazar O Buchwald P. Design, synthesis, and evaluation of novel immunomodulatory small molecules targeting the CD40-CD154 costimulatory protein-protein interaction. Molecules. (2018) 23:1153. doi: 10.3390/molecules23051153 29751636 PMC5978685

[89] Abdulreda MH Caicedo A Berggren P-O. Transplantation into the anterior chamber of the eye for longitudinal, non-invasive in *vivo* imaging with single-cell resolution in real-time. J Vis Exp. (2013) 73):e50466. doi: 10.3791/50466 PMC3633237 23524511

[90] Abdulreda MH Faleo G Molano RD Lopez-Cabezas M Molina J Tan Y. High-resolution, noninvasive longitudinal live imaging of immune responses. Proc Natl Acad Sci USA. (2011) 108:12863–8. doi: 10.1073/pnas.1105002108 PMC3150878 21768391

[91] Miska J Abdulreda MH Devarajan P Lui JB Suzuki J Pileggi A. Real-time immune cell interactions in target tissue during autoimmune-induced damage and graft tolerance. J Exp Med. (2014) 211:441–56. doi: 10.1084/jem.20130785 PMC3949570 24567447

[92] Abdulreda MH Molano RD Faleo G Lopez-Cabezas M Shishido A Ulissi U. *In vivo* imaging of type 1 diabetes immunopathology using eye-transplanted islets in NOD mice. Diabetologia. (2019) 62:1237–50. doi: 10.1007/s00125-019-4879-0 PMC6561836 31087105

[93] Buchwald P Juhász A Bell C Pátfalusi M Howes J. Bodor N. A pharmacogenetics-based unified parent-metabolite pharmacokinetic model incorporating acetylation polymorphism for talampanel in humans. J Pharmacokinet Pharmacodyn. (2005) 32:377–400. doi: 10.1007/s10928-005-0001-y 16320099

[94] Nanji SA Hancock WW Luo B Schur CD Pawlick RL Zhu LF. Costimulation blockade of both inducible costimulator and CD40 ligand induces dominant tolerance to islet allografts and prevents spontaneous autoimmune diabetes in the NOD mouse. Diabetes. (2006) 55:27–33. doi: 10.2337/diabetes.55.01.06.db04-1154 16380473

[95] Buchwald P. Structure-metabolism relationships: steric effects and the enzymatic hydrolysis of carboxylic esters. Mini-Rev Med Chem. (2001) 1:101–11. doi: 10.2174/1389557013407403 12369995

[96] Bluestone JA Herold K Eisenbarth G. Genetics, pathogenesis and clinical interventions in type 1 diabetes. Nature. (2010) 464:1293–300. doi: 10.1038/nature08933 PMC4959889 20432533

[97] Atkinson MA Eisenbarth GS Michels AW. Type 1 diabetes. Lancet. (2014) 383:69–82. doi: 10.1016/s0140-6736(13)60591-7 23890997 PMC4380133

[98] Lemos JRN Hirani K von Herrath M. Immunological and virological triggers of type 1 diabetes: insights and implications. Front Immunol. (2023) 14:2023.1326711. doi: 10.3389/fimmu.2023.1326711 PMC10794398 38239343

[99] Quattrin T Mastrandrea LD Walker LSK. Type 1 diabetes. Lancet. (2023) 401:2149–62. doi: 10.1016/s0140-6736(23)00223-4 37030316

[100] Green A Hede SM Patterson CC Wild SH Imperatore G Roglic G. Type 1 diabetes in 2017: global estimates of incident and prevalent cases in children and adults. Diabetologia. (2021) 64:2741–50. doi: 10.1007/s00125-021-05571-8 PMC8563635 34599655

[101] Gregory GA Robinson TIG Linklater SE Wang F Colagiuri S de Beaufort C. Global incidence, prevalence, and mortality of type 1 diabetes in 2021 with projection to 2040: a modelling study. Lancet Diabetes Endocrinol. (2022) 10:741–60. doi: 10.1016/s2213-8587(22)00218-2 36113507

[102] Fang M Wang D Selvin E. Prevalence of type 1 diabetes among US children and adults by age, sex, race, and ethnicity. J Am Med Assoc (JAMA). (2024) 331:1411–3. doi: 10.1001/jama.2024.2103 PMC11040401 38573653

[103] Pambianco G Costacou T Ellis D Becker DJ Klein R Orchard TJ. The 30-year natural history of type 1 diabetes complications: the Pittsburgh Epidemiology of Diabetes Complications Study experience. Diabetes. (2006) 55:1463–9. doi: 10.2337/db05-1423 16644706

[104] Gregg EW Li Y Wang J Burrows NR Ali MK Rolka D. Changes in diabetes-related complications in the United States, 1990-2010. N Engl J Med. (2014) 370:1514–23. doi: 10.1056/NEJMoa1310799 24738668

[105] Zheng Y Ma Q Qi X Zhu Z Wu B. Prevalence and incidence of mild cognitive impairment in adults with diabetes in the United States. Diabetes Res Clin Pract. (2023) 205:110976. doi: 10.1016/j.diabres.2023.110976 37890703

[106] Livingstone SJ Levin D Looker HC Lindsay RS Wild SH Joss N. Estimated life expectancy in a Scottish cohort with type 1 diabetes, 2008-2010. J Am Med Assoc (JAMA). (2015) 313:37–44. doi: 10.1001/jama.2014.16425 PMC4426486 25562264

[107] Rawshani A Sattar N Franzen S Rawshani A Hattersley AT Svensson AM. Excess mortality and cardiovascular disease in young adults with type 1 diabetes in relation to age at onset: a nationwide, register-based cohort study. Lancet. (2018) 392:477–86. doi: 10.1016/s0140-6736(18)31506-x PMC6828554 30129464

[108] von Scholten BJ Kreiner FF Gough SCL von Herrath M. Current and future therapies for type 1 diabetes. Diabetologia. (2021) 64:1037–48. doi: 10.1007/s00125-021-05398-3 PMC8012324 33595677

[109] Dabelea D Mayer-Davis EJ Saydah S Imperatore G Linder B Divers J. Prevalence of type 1 and type 2 diabetes among children and adolescents from 2001 to 2009. J Am Med Assoc (JAMA). (2014) 311:1778–86. doi: 10.1001/jama.2014.3201 PMC4368900 24794371

[110] Patterson CC Harjutsalo V Rosenbauer J Neu A Cinek O Skrivarhaug T. Trends and cyclical variation in the incidence of childhood type 1 diabetes in 26 European centres in the 25 year period 1989-2013: a multicentre prospective registration study. Diabetologia. (2019) 62:408–17. doi: 10.1007/s00125-018-4763-3 30483858

[111] Wagenknecht LE Lawrence JM Isom S Jensen ET Dabelea D Liese AD. Trends in incidence of youth-onset type 1 and type 2 diabetes in the USA, 2002-18: results from the population-based SEARCH for Diabetes in Youth study. Lancet Diabetes Endocrinol. (2023) 11:242–50. doi: 10.1016/s2213-8587(23)00025-6 PMC10091237 36868256

[112] Lerner A Jeremias P Matthias T. The world incidence and prevalence of autoimmune diseases is increasing. Int J Celiac Dis. (2015) 3:151–5. doi: 10.12691/ijcd-3-4-8

[113] Skyler JS Ricordi C. Stopping type 1 diabetes: attempts to prevent or cure type 1 diabetes in man. Diabetes. (2011) 60:1–8. doi: 10.2337/db10-1114 21193733 PMC3012160

[114] Skyler JS. Prevention and reversal of type 1 diabetes - past challenges and future opportunities. Diabetes Care. (2015) 38:997–1007. doi: 10.2337/dc15-0349 25998292

[115] Jacobsen LM Schatz D. Immunotherapy-based strategies for treatment of type 1 diabetes. Horm Res Paediatr. (2024) 97:ePub. doi: 10.1159/000542002 PMC12038710 39401495

[116] Hirsch JS. FDA approves teplizumab: a milestone in type 1 diabetes. Lancet Diabetes Endocrinol. (2023) 11:18. doi: 10.1016/s2213-8587(22)00351-5 36436528

[117] Herold KC Bundy BN Long SA Bluestone JA DiMeglio LA Dufort MJ. An anti-CD3 antibody, teplizumab, in relatives at risk for type 1 diabetes. N Engl J Med. (2019) 381:603–13. doi: 10.1056/nejmoa1902226 PMC6776880 31180194

[118] Sims EK Bundy BN Stier K Serti E Lim N Long SA. Teplizumab improves and stabilizes beta cell function in antibody-positive high-risk individuals. Sci Trans Med. (2021) 13:eabc8980. doi: 10.1126/scitranslmed.abc8980 PMC8610022 33658358

[119] Bluestone JA Buckner JH Herold KC. Immunotherapy: building a bridge to a cure for type 1 diabetes. Science. (2021) 373:510–6. doi: 10.1126/science.abh1654 34326232

[120] Vincenti F Luggen M. T cell costimulation: a rational target in the therapeutic armamentarium for autoimmune diseases and transplantation. Annu Rev Med. (2007) 58:347–58. doi: 10.1146/annurev.med.58.080205.154004 17020493

[121] Sharpe AH. Mechanisms of costimulation. Immunol Rev. (2009) 229:5–11. doi: 10.1111/j.1600-065x.2009.00784.x 19426211 PMC2928676

[122] Li XC Rothstein DM Sayegh MH. Costimulatory pathways in transplantation: challenges and new developments. Immunol Rev. (2009) 229:271–93. doi: 10.1111/j.1600-065X.2009.00781.x PMC13741320 19426228

[123] Nir T Melton DA Dor Y. Recovery from diabetes in mice by β cell regeneration. J Clin Invest. (2007) 117:2553–61. doi: 10.1172/jci32959 PMC1957545 17786244

[124] Zahr E Molano RD Pileggi A Ichii H Jose SS Bocca N. Rapamycin impairs in *vivo* proliferation of islet beta-cells. Transplantation. (2007) 84:1576–83. doi: 10.1097/01.tp.0000296035.48728.28 18165767

[125] Shapiro AM Pokrywczynska M Ricordi C. Clinical pancreatic islet transplantation. Nat Rev Endocrinol. (2017) 13:268–77. doi: 10.1038/nrendo.2016.178 27834384

[126] Lablanche S Vantyghem MC Kessler L Wojtusciszyn A Borot S Thivolet C. Islet transplantation versus insulin therapy in patients with type 1 diabetes with severe hypoglycaemia or poorly controlled glycaemia after kidney transplantation (TRIMECO): a multicentre, randomised controlled trial. Lancet Diabetes Endocrinol. (2018) 6:527–37. doi: 10.1016/S2213-8587(18)30078-0 29776895

[127] Vantyghem MC Chetboun M Gmyr V Jannin A Espiard S Le Mapihan K. Ten-year outcome of islet alone or islet after kidney transplantation in type 1 diabetes: A prospective parallel-arm cohort study. Diabetes Care. (2019) 42:2042–9. doi: 10.2337/dc19-0401 31615852

[128] Markmann JF Rickels MR Eggerman TL Bridges ND Lafontant DE Qidwai J. Phase 3 trial of human islet-after-kidney transplantation in type 1 diabetes. Am J Transplant. (2021) 21:1477–92. doi: 10.1111/ajt.16174 PMC9074710 32627352

[129] Stabler CL Russ HA. Regulatory approval of islet transplantation for treatment of type 1 diabetes: Implications and what is on the horizon. Mol Ther. (2023) 31:3107–8. doi: 10.1016/j.ymthe.2023.10.011 PMC10638039 37865099

[130] Johnson JD. The quest to make fully functional human pancreatic beta cells from embryonic stem cells: climbing a mountain in the clouds. Diabetologia. (2016) 59:2047–57. doi: 10.1007/s00125-016-4059-4 27473069

[131] Siehler J Blochinger AK Meier M Lickert H. Engineering islets from stem cells for advanced therapies of diabetes. Nat Rev Drug Discovery. (2021) 20:920–40. doi: 10.1038/s41573-021-00262-w 34376833

[132] de Koning EJP Carlotti F. Stem cell-based islet replacement therapy in diabetes: A road trip that reached the clinic. Cell Stem Cell. (2021) 28:2044–6. doi: 10.1016/j.stem.2021.11.008 34861145

[133] The Lancet Diabetes and Endocrinology. Stem-cell therapy for diabetes: the hope continues. Lancet Diabetes Endocrinol. (2024) 12:775. doi: 10.1016/S2213-8587(24)00314-0 39396525

[134] Mojibian M Harder B Hurlburt A Bruin JE Asadi A Kieffer TJ. Implanted islets in the anterior chamber of the eye are prone to autoimmune attack in a mouse model of diabetes. Diabetologia. (2013) 56:2213–21. doi: 10.1007/s00125-013-3004-z 23933952

[135] Chmelova H Cohrs CM Chouinard JA Petzold C Kuhn M Chen C. Distinct roles of beta-cell mass and function during type 1 diabetes onset and remission. Diabetes. (2015) 64:2148–60. doi: 10.2337/db14-1055 25605805

[136] Schmidt-Christensen A Hansen L Ilegems E Fransen-Pettersson N Dahl U Gupta S. Imaging dynamics of CD11c(+) cells and Foxp3(+) cells in progressive autoimmune insulitis in the NOD mouse model of type 1 diabetes. Diabetologia. (2013) 56:2669–78. doi: 10.1007/s00125-013-3024-8 23963325

[137] Berclaz C Schmidt-Christensen A Szlag D Extermann J Hansen L Bouwens A. Longitudinal three-dimensional visualisation of autoimmune diabetes by functional optical coherence imaging. Diabetologia. (2016) 59:550–9. doi: 10.1007/s00125-015-3819-x 26613896

[138] Benson RA Garcon F Recino A Ferdinand JR Clatworthy MR Waldmann H. Non-invasive multiphoton imaging of islets transplanted into the pinna of the NOD mouse ear reveals the immediate effect of anti-CD3 treatment in autoimmune diabetes. Front Immunol. (2018) 9:2018.01006. doi: 10.3389/fimmu.2018.01006 29867981 PMC5968092

[139] Ilegems E Dicker A Speier S Sharma A Bahow A Edlund PK. Reporter islets in the eye reveal the plasticity of the endocrine pancreas. Proc Natl Acad Sci USA. (2013) 110:20581–6. doi: 10.1073/pnas.1313696110 PMC3870708 24248353

[140] Ellis CE Mojibian M Ida S Fung VCW Skovso S McIver E. Human A2-CAR T cells reject HLA-A2 + human islets transplanted into mice without inducing graft-versus-host disease. Transplantation. (2023) 107:e222–e33. doi: 10.1097/tp.0000000000004709 PMC10527662 37528526

[141] Makino S Kunimoto K Muraoka Y Mizushima Y Katagiri K Tochino Y. Breeding of a non-obese, diabetic strain of mice. Jikken Dobutsu (Exp Anim). (1980) 29:1–13. doi: 10.1538/expanim1978.29.1_1 6995140

[142] Leiter EH. The NOD mouse: a model for insulin-dependent diabetes mellitus. Curr Protoc Immunol. (2001) 15:9.1–.9.23. doi: 10.1002/0471142735.im1509s24 18432739

[143] Roep BO Atkinson M von Herrath M. Satisfaction (not) guaranteed: re-evaluating the use of animal models of type 1 diabetes. Nat Rev Immunol. (2004) 4:989–97. doi: 10.1038/nri1502 15573133

[144] Anderson MS Bluestone JA. The NOD mouse: a model of immune dysregulation. Annu Rev Immunol. (2005) 23:447–85. doi: 10.1146/annurev.immunol.23.021704.115643 15771578

[145] Shoda LK Young DL Ramanujan S Whiting CC Atkinson MA Bluestone JA. A comprehensive review of interventions in the NOD mouse and implications for translation. Immunity. (2005) 23:115–26. doi: 10.1016/j.immuni.2005.08.002 16111631

[146] Leiter EH SChile A. Genetic and pharmacologic models for type 1 diabetes. Curr Protoc Mouse Biol. (2013) 3:9–19. doi: 10.1002/9780470942390.mo120154 24592352 PMC3936677

[147] Reed JC Herold KC. Thinking bedside at the bench: the NOD mouse model of T1DM. Nat Rev Endocrinol. (2015) 11:308–14. doi: 10.1038/nrendo.2014.236 PMC4523382 25623120

