# Supplementary material for: Small-molecule inhibitors of the CD40–CD40L costimulatory interaction are effective in pancreatic islet transplantation and prevention of type 1 diabetes models
Source: Front Immunol. 2024 Nov 13;15:1484425. doi: 10.3389/fimmu.2024.1484425 (PMC11599200; doi:10.3389/fimmu.2024.1484425)
Supplement: Supplementary file 1 [file DataSheet1.pdf]

Supplementary Material

Small-Molecule Inhibitors of the CD40–CD40L Costimulatory Interaction Are Effective in Pancreatic Islet Transplantation and Prevention of Type 1 Diabetes Models

Sung-Ting Chuang[1], Oscar Alcazar[1], Brandon Watts[1], Midhat H. Abdulreda*[1,2,3,4], and Peter Buchwald*[1,5]

[1]Diabetes Research Institute, Miller School of Medicine, University of Miami, Miami, Florida, USA

[2]Department of Surgery, Miller School of Medicine, University of Miami, Miami, Florida, USA

[3]Department of Microbiology and Immunology, Miller School of Medicine, University of Miami, Miami, Florida, USA

[4]Department of Ophthalmology, Miller School of Medicine, University of Miami, Miami, Florida, USA

[5]Department of Molecular and Cellular Pharmacology, Miller School of Medicine, University of Miami, Miami, Florida, USA

1 Supplementary Figures and Tables

- Supplementary Figure 1. Comparison of immune cell populations in NOD mouse splenocytes.

1.1 Supplementary Figures

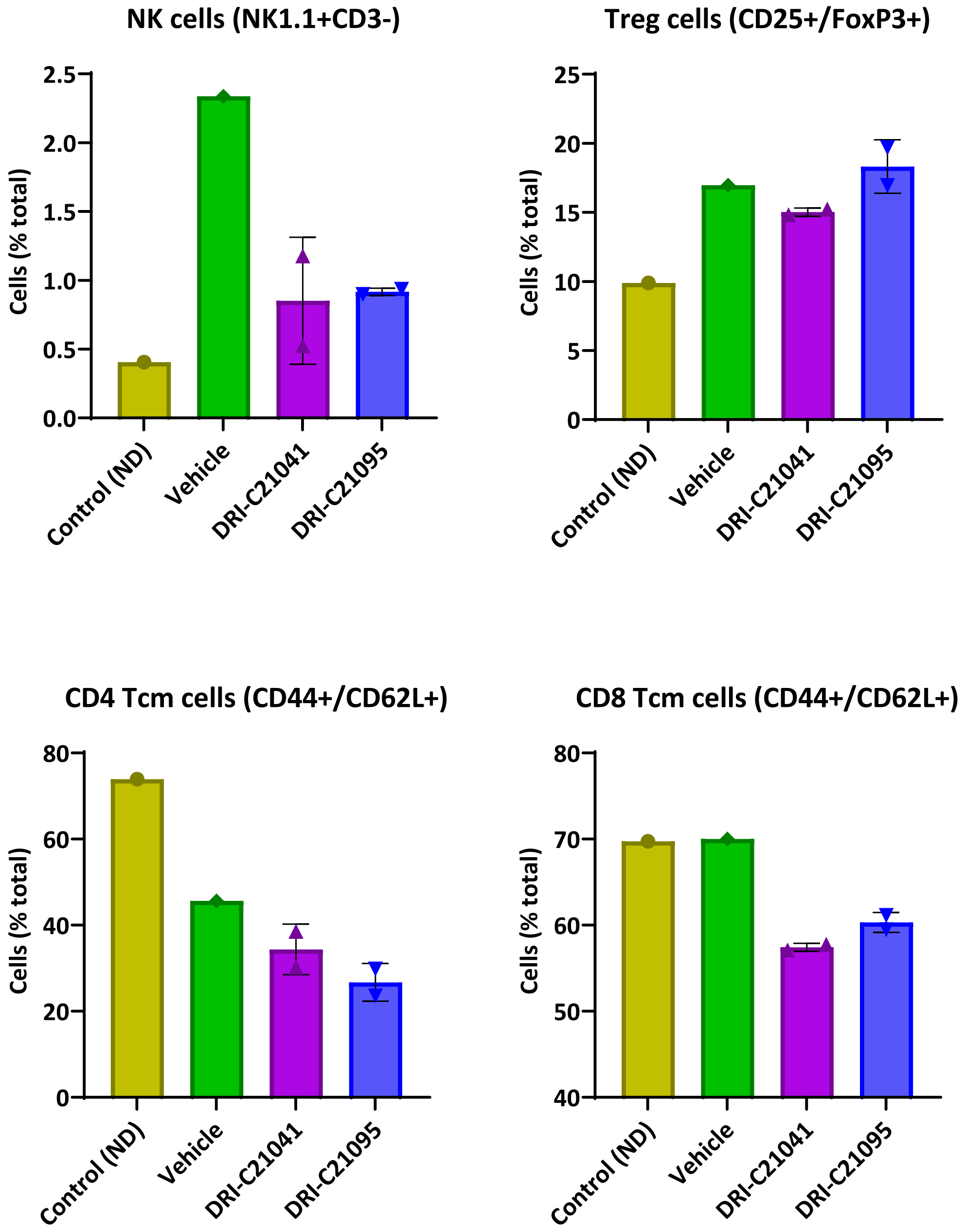

Supplementary Figure 1. Comparison of immune cell populations in NOD mouse splenocytes. Percent of cells shown in 40-weeks old NOD mice that were treated with DRI-C21041 (purple) or DRI-C21095 (blue) during weeks 5–18 and remained diabetes free ($n = 2$) compared to those in a vehicle-treated control NOD that also remained diabetes free until the same age (green) and a younger not yet diabetic naïve control NOD (mustard). Data shown are the percentages of each cell subset among total live splenocytes with noticeable differences at this age as determined by flow cytometry: NK ($NK1.1^+CD3^-$), Treg ($CD25^+FoxP3^+$, % of total CD3CD4), CD4 Tcm ($CD44^+CD62L^+$, % of total CD3CD4), and CD8 Tcm ($CD44^+CD62L^+$, % of total CD3CD8).
